# Supplementary material for: Luminescent Trityl‐based Diradicaloids: A Theoretical and Experimental Assessment of Charge‐Resonance in Low‐Lying Excited States
Source: Chemistry. 2025 Mar 24;31(23):e202500749. doi: 10.1002/chem.202500749 (PMC12015404; doi:10.1002/chem.202500749)
Supplement: Supplementary file 1 — Supporting Information [file CHEM-31-e202500749-s001.pdf]

Chemistry–A European Journal

Supporting Information

Luminescent Trityl-based Diradicaloids: A Theoretical and Experimental Assessment of Charge-Resonance in Low-Lying Excited States

Davide Mesto, Michele Orza, Brunella Bardi, Angela Punzi, Imma Ratera, Jaume Veciana, Gianluca Farinola, Anna Painelli, Francesca Terenziani,* Davide Blasi,* and Fabrizia Negri*

Supporting Information

Luminescent Trityl-based Diradicaloids: A Theoretical and Experimental Assessment of Charge-Resonance in Low-Lying Excited States

Davide Mesto,[a,‡] Michele Orza,[b,‡] Brunella Bardi,[c] Angela Punzi,[a] Imma Ratera,[d] Jaume Veciana,[d] Gianluca Farinola,[a] Anna Painelli,[c] Francesca Terenziani*,[c] Davide Blasi*,[a] Fabrizia Negri*,[b,e,f]

a) Dipartimento di Chimica, Università degli Studi di Bari Aldo Moro, 70125 – Bari, Italy
b) Dipartimento di Chimica "Giacomo Ciamician", Università di Bologna, 40129 – Bologna, Italy
c) Dipartimento di Scienze Chimiche, della Vita e della Sostenibilità Ambientale, Università di Parma, Parco Area delle Scienze 17/a, 43124 Parma, Italy
d) Institut de Ciencia de Materials de Barcelona (CSIC)/CIBER-BBN, Campus de la UAB, 08193 -Bellaterra, Barcelona, Spain.
e) INSTM UdR Bologna, 40129 Bologna, Italy.
f) Center for Chemical Catalysis—C3, Università di Bologna, 40129 Bologna, Italy

‡These authors equally contributed

Contents

1. Synthesis 2
2. Computational details 4
 2.1. Fragment orbital approach. 12
 2.2. Diradical character $y0$. 17
 2.3. Identification of the SE and DE states from TDUDFT calculations. 17
3. Experimental details 29
4. Cartesian coordinates of optimized geometries 31

1. Synthesis

General Remarks: Tetrahydrofuran was distilled from sodium/benzophenone before use. $CHCl_3$ and CH_2Cl_2 used for purification of **TTM-TTM** were percolated on basic alumina. Other solvents and chemicals were purchased at the highest commercial purity and used without further purification. Preparative column chromatography was performed using Macherey-Nagel silica gel (60, particle size 0.040-0.063 mm). Macherey-Nagel aluminium sheets with silica gel 60 F254 were used for TLC. ^{1}H NMR spectra were acquired on an Agilent 500 spectrometer at 500 MHz using the $CDCl_3$ residual proton peak at δ = 7.26 ppm as internal standard. High-resolution mass spectra were acquired with a Shimadzu high-performance liquid chromatography ion trap time-of-flight (LC-IT-TOF) mass spectrometer *via* direct infusion of the samples by using methanol as the elution solvent (the samples were previously dissolved in THF, and few drops of the THF solution were added to methanol).

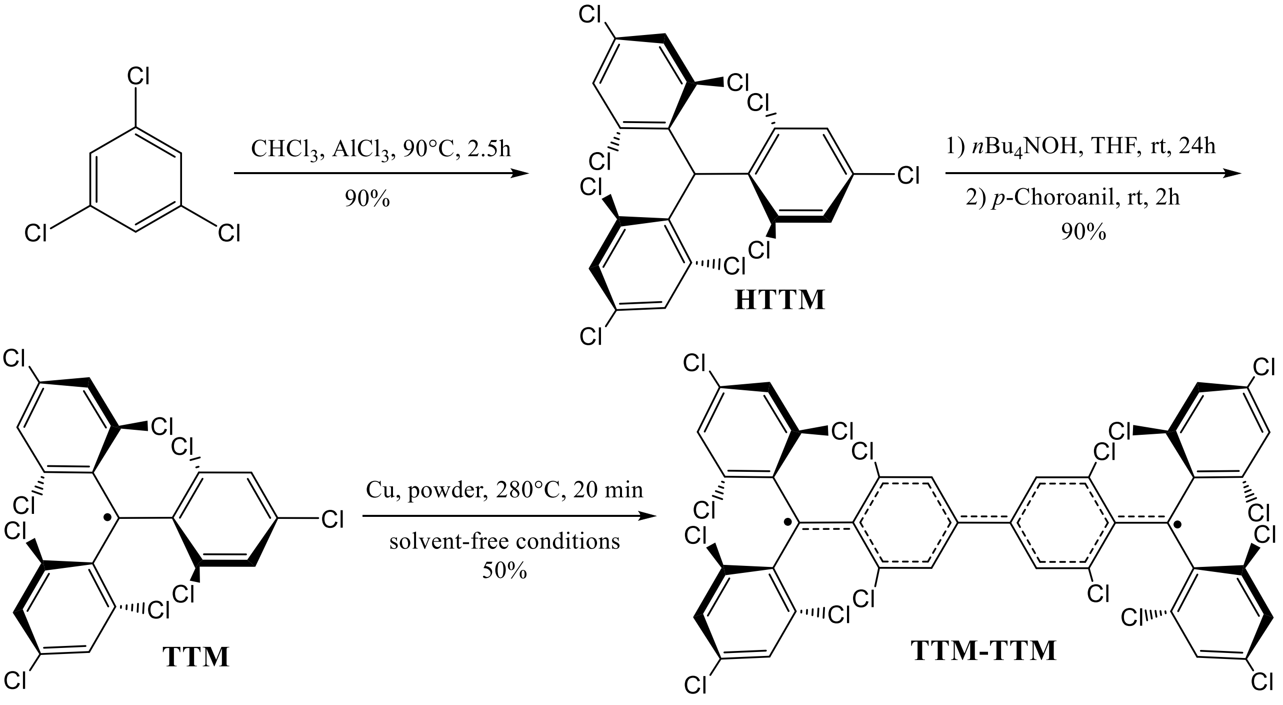

Scheme S1: Three-steps synthesis of **TTM-TTM**.

Synthesis of Tris(2,4,6-trichlorophenyl)methane HTTM:[1,2] A 50 mL two-necked round bottom flask, equipped with a reflux condenser and a magnetic stirrer, was charged with 1,3,5-trichlorobenzene (3.00 g, 16.53 mmol), $AlCl_3$ (245 mg, 1.84 mmol). This mixture was heated to 90 °C, then chloroform (150 µL, 1.84 mmol) was added. The reaction mixture was stirred for 2.5 h at 90°C, then it was cooled to room temperature, quenched with a 1N HCl solution and extracted with $CHCl_3$ (3x50mL). The combined organic phases were washed with brine, dried over anhydrous Na_2SO_4 and the solvent was removed under vacuum. The crude product (brownish solid) was washed three times with hot hexane affording pure **HTTM** as a white solid (1.08 g, 90% yield). ^{1}HNMR data agree with those previously reported in the literature.[1b] ^{1}H NMR (500 MHz, $CDCl_3$) δ: 7.36 (d, J = 2.3 Hz, 3H), 7.24 (d, J = 2.3 Hz, 3H), 6.68 (s, 1H).

Synthesis of Tris(2,4,6-trichlorophenyl)methane radical TTM:[2] A 100 mL three-necked round bottom flask, covered with aluminium foil and equipped with a magnetic stirrer, was charged with a solution of **HTTM** (500 mg, 0.90 mmol) in 50 mL of anhydrous THF, under nitrogen atmosphere. A 56% w/w aqueous solution of tetrabutylammonium hydroxide (546 µL, 1.17 mmol) was added to the reaction mixture that became immediately orange. The resulting mixture stirred for 24 h at room temperature in the dark, then tetrachloro-1,4-benzoquinone (310 mg, 1.26 mmol) was added. After stirring for 2h, the reaction solvent

was removed at reduced pression and the crude product was purified by column chromatography (silica gel, hexane/dichloromethane 8:2) to give **TTM** as an orange solid (450 mg, yield 90%).

Synthesis of TTM-TTM (best conditions, entry 8 of **Table S1**): A 15 mL pressure tube, equipped with a zirconia ball (10 mm diameter used as a stirrer), was charged with **TTM** (100 mg, 0.18 mmol) in presence of and Cu powder (115 mg, 1.8 mmol). The reactor was placed in rotation and heated at 280 °C using a Büchi drying oven for 20 minutes, then it was cooled to room temperature. The reaction mixture was diluted in $CHCl_3$, then filtered to remove Cu powder. The solvent was removed under vacuum, then the crude product was purified by two subsequent chromatographic columns: a) (silica gel, hexane:CH_2Cl_2 = 8:2) to remove unreacted **TTM**; (silica gel, hexane:$CHCl_3$ = 9:1) to remove more polar impurities. 45 mg of **TTM-TTM** as a blue solid were obtained (50% yield). HRMS (LC-IT-TOF, elution with methanol) *m/z*: M^- calculated for $C_{38}H_{12}Cl_{16}$ 1027.5961; found 1027.5989.

Table S1. Experimental conditions for **TTM-TTM** synthesis.

Entry	Cu [mol%]	Temperature [°C]	Time [min]	Yield %
1	0	280	20	5
2	0	280	30	10
3	0	280	45	8
4	0	280	60	7
5	5	280	30	10
6	10	200	90	0[a]
7	10	250	90	0[a]
**8**	**10**	280	**20**	**50**
9	10	280	30	20
10	30	280	120	0[b]

a) **TTM** was completely recovered. b) Formation of several different subproducts showing a lower retention factor compared to **TTM-TTM**.

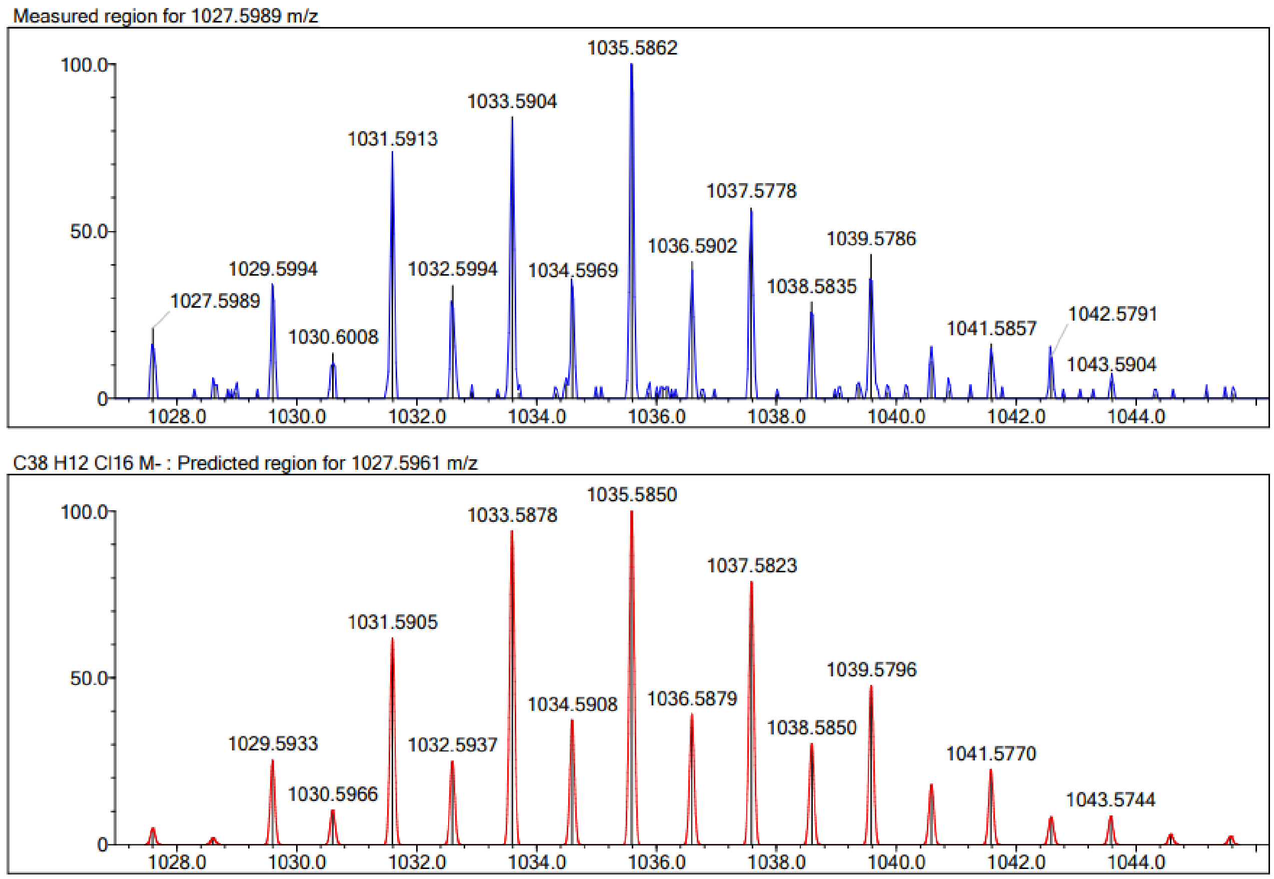

Figure S1. HRMS for **TTM-TTM**: top) intensity distribution for the signals in the region 1027.5989 m/z; bottom) predicted intensity distribution for the signals in the region 1027.5961 m/z.

Thermal gravimetric analysis (TGA) was carried out on a Pyris 1-Perkin Elmer thermogravimetric analysis system, applying a nitrogen flow of 40 mL/min. TTM and TTM-TTM were maintained at 100°C for 3 min and then they were heated at a rate of 5°C min^{-1} in the range of temperature 100-550°C.

2. Computational details

Three diradicals based on **TTM** were considered: **TTM-TTM** and **TTM-ph-TTM** corresponding to experimentally investigated systems and a longer member in the series, **TTM-ph-ph-TTM**. Quantum-chemical calculations were carried out using density functional theory (DFT) for the ground electronic state and time-dependent DFT (TDDFT) for excited states. Singlet spin ground state geometries were determined at restricted (RDFT) and unrestricted (UDFT) level, employing the M062X/def2SVP with the Grimme dispersion contribution D3[3,4] The UDFT optimized geometries are characterized by localized broken symmetry (BS) orbitals. Such optimized geometries are labelled in the following as the BS geometries. TDDFT and TDUDFT calculations were carried out at the ground state geometries either employing the M062X functional and the B3LYP functional. The latter has been previously shown to provide acceptably small spin contamination at unrestricted level and to describe satisfactorily the DE state of conjugated diradicals displaying large diradical character[5–7]. Furthermore, the TDUB3LYP calculations on the **TTM** radical showed closer agreement with the experimental absorption spectrum (see Figure S2 and Tables S2-S3). Notably, TDUM062X calculations, are affected by much larger spin-contamination and strongly overestimate the excitation energy of the lowest excited states in these

diradicals. For the above reasons, the discussion on the lowest lying excited states of the diradicals investigated, was based on TDUB3LYP results.

Beside DFT and TDDFT calculations, the excitation energies were determined with the complete active space self-consistent field (CASSCF)[8] calculations, followed by NEVPT2[9,10] corrections to include dynamical correlation. Most calculations were carried out with an active space including 4 electrons in 3 orbitals CASSCF(4,3). The optimized geometry of the ground and DE excited states were also determined at CASSCF(4,3) level. A graphical representation of UDFT optimized and CASSCF optimized ground state geometries is collected in Figures S3-S5. All CASSCF calculations were performed with ORCA 5.0.4[11,12] using the def2-SVP basis set.[13] The resolution of identity approximation and the related basis sets for both Coulomb and HF exchange integrals were used (RI-JK).[14] Three roots were calculated for the state averaged modelling the singlet excited states.

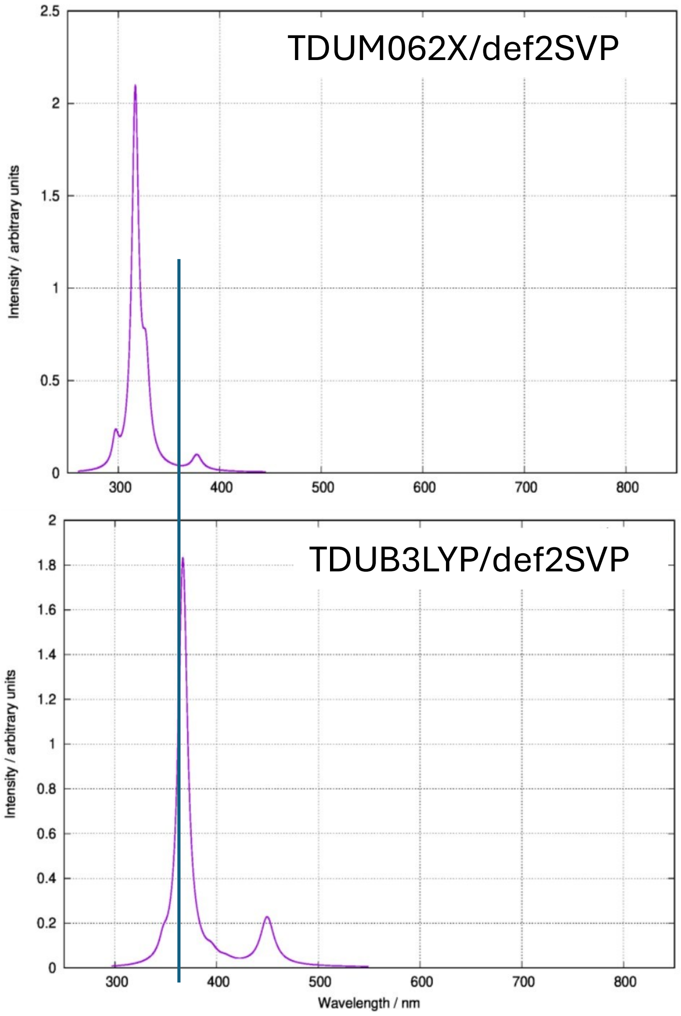

Figure S2. Simulated absorption spectrum of **TTM** radical at TDUM062X/def2SVP +D3 level (top) and TDUB3LYP/def2SVP +D3 level (bottom) and comparison with the main absorption band in the exp spectrum (blue vertical line). The comparison shows that TDUB3LYP/def2SVP calculations are in better agreement with experimental results.

Table S2. Excited states of **TTM** radical from TDUB3LYP/def2SVP +D3 calculations @ optimized UM062X/def2SVP +D3 ground state geometry.

```
Excitation energies and oscillator strengths:
 Excited State   1:  2.259-E      2.7577 eV  449.59 nm  f=0.0169  <S**2>=1.026
    137A ->138A        0.30573
    134B ->137B        0.28511
    136B ->137B        0.85635
 Excited State   2:  2.259-E      2.7577 eV  449.59 nm  f=0.0169  <S**2>=1.026
    137A ->139A        0.30573
    133B ->137B       -0.28511
    135B ->137B        0.85635
 Excited State   3:  2.141-E      3.0319 eV  408.93 nm  f=0.0010  <S**2>=0.896
    137A ->138A        0.20418
    134B ->137B        0.87042
    136B ->137B       -0.39465
 Excited State   4:  2.141-E      3.0319 eV  408.93 nm  f=0.0010  <S**2>=0.896
    137A ->139A       -0.20419
    133B ->137B        0.87042
    135B ->137B        0.39465
 Excited State   5:  2.391-A1     3.0805 eV  402.48 nm  f=0.0000  <S**2>=1.180
    133A ->139A       -0.11621
    134A ->138A       -0.11621
    135A ->139A       -0.14705
    136A ->138A       -0.14704
    137A ->143A       -0.23739
    125B ->137B        0.10010
    131B ->137B        0.86906
    132B ->140B       -0.11056
 Excited State   6:  2.122-A2     3.1406 eV  394.78 nm  f=0.0051  <S**2>=0.876
    137A ->140A       -0.22832
    132B ->137B        0.95819
 Excited State   7:  2.441-E      3.3813 eV  366.68 nm  f=0.1434  <S**2>=1.239
    132A ->142A        0.12259
    133A ->140A       -0.13202
    137A ->138A        0.76494
    131B ->138B        0.13317
    134B ->137B       -0.35640
    135B ->139B        0.11096
    136B ->137B       -0.28769
    136B ->138B       -0.11096
 Excited State   8:  2.441-E      3.3813 eV  366.68 nm  f=0.1434  <S**2>=1.239
    132A ->141A        0.12259
    134A ->140A        0.13202
    137A ->139A        0.76494
    131B ->139B        0.13317
    133B ->137B        0.35640
    135B ->137B       -0.28768
    135B ->138B        0.11096
    136B ->139B        0.11096
```

Table S3. Excited states of **TTM** radical from TDUM062X/def2SVP +D3 calculations @ optimized UM062X/def2SVP + D3 ground state geometry.

```
Excited State   1:  2.457-E      3.2858 eV  377.34 nm  f=0.0065  <S**2>=1.259
    135A ->139A       -0.10375
    136A ->138A       -0.10375
    137A ->139A        0.42537
    130B ->137B       -0.11142
    131B ->139B       -0.16718
    133B ->137B       -0.34632
    135B ->137B        0.71549
    135B ->138B        0.11437
    136B ->139B        0.11437

 Excited State   2:  2.457-E      3.2858 eV  377.33 nm  f=0.0065  <S**2>=1.259
    135A ->138A        0.10378
    136A ->139A       -0.10372
    137A ->138A        0.42542
    129B ->137B       -0.11140
    131B ->138B       -0.16717
    134B ->137B        0.34631
    135B ->139B        0.11434
    136B ->137B        0.71549
    136B ->138B       -0.11438

 Excited State   3:  2.545-A1     3.5030 eV  353.94 nm  f=0.0000  <S**2>=1.369
    133A ->138A        0.13688
    134A ->139A        0.13694
    135A ->138A        0.16568
    136A ->139A        0.16580
    137A ->143A        0.26489
    125B ->137B       -0.16438
    131B ->137B        0.79991
    132B ->140B        0.10715
    133B ->139B        0.12900
    134B ->138B       -0.12895
    135B ->139B       -0.15531
    136B ->138B       -0.15519

 Excited State   4:  2.335-E      3.7892 eV  327.21 nm  f=0.0377  <S**2>=1.113
    135A ->140A        0.11094
    137A ->139A       -0.49586
    137A ->142A       -0.12608
    132B ->138B        0.15546
    133B ->137B        0.48845
    135B ->137B        0.60092
    135B ->138B       -0.10125
    136B ->139B       -0.10123

 Excited State   5:  2.335-E      3.7892 eV  327.21 nm  f=0.0376  <S**2>=1.113
    136A ->140A       -0.11093
    137A ->138A       -0.49572
    137A ->141A        0.12612
    132B ->139B       -0.15549
    134B ->137B       -0.48859
    135B ->139B       -0.10122
    136B ->137B        0.60091
    136B ->138B        0.10127

 Excited State   6:  2.420-A2     3.8811 eV  319.45 nm  f=0.0019  <S**2>=1.215
    135A ->142A        0.11388
    136A ->141A        0.11390
    137A ->140A       -0.51637
    132B ->137B        0.74671
```

```
   133B ->138B        0.16442
   134B ->139B        0.16446
   135B ->138B        0.13257
   136B ->139B       -0.13259

Excited State   7:  2.267-E      3.9160 eV  316.61 nm  f=0.1584  <S**2>=1.035
   133A ->140A        0.10132
   137A ->139A        0.58719
   137A ->142A       -0.19877
   130B ->137B        0.11982
   132B ->138B        0.12013
   133B ->137B        0.68313
   135B ->137B       -0.12048

Excited State   8:  2.267-E      3.9161 eV  316.60 nm  f=0.1585  <S**2>=1.035
   134A ->140A        0.10133
   137A ->138A       -0.58726
   137A ->141A       -0.19876
   129B ->137B       -0.11983
   132B ->139B        0.12009
   134B ->137B        0.68304
   136B ->137B        0.12065
```

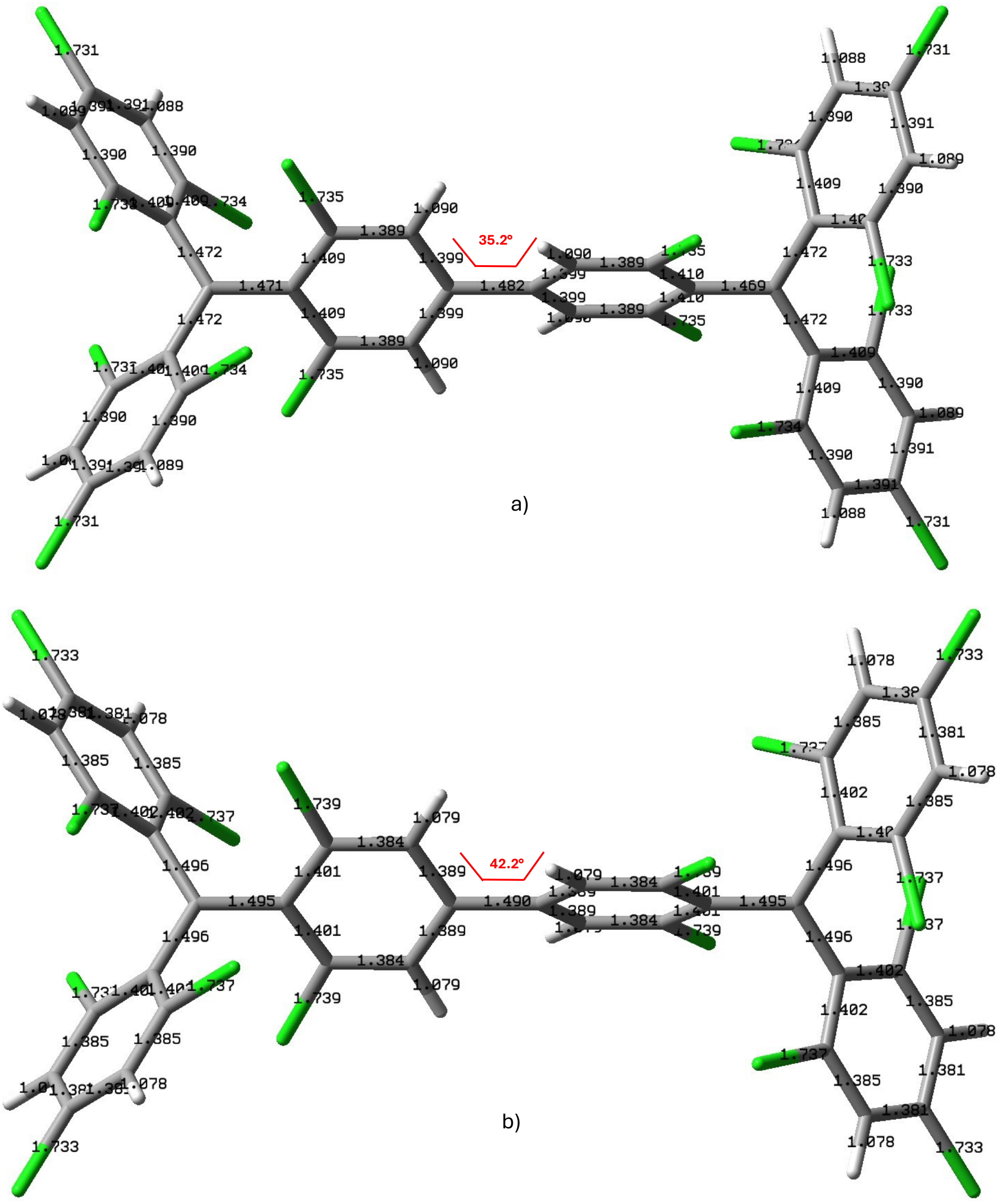

Figure S3. Computed bond lengths (Å) of the ground state of **TTM-TTM** at (a) UM06-2X/def2SVP +D3 level and (b) CAS(4,3)/def2SVP level.

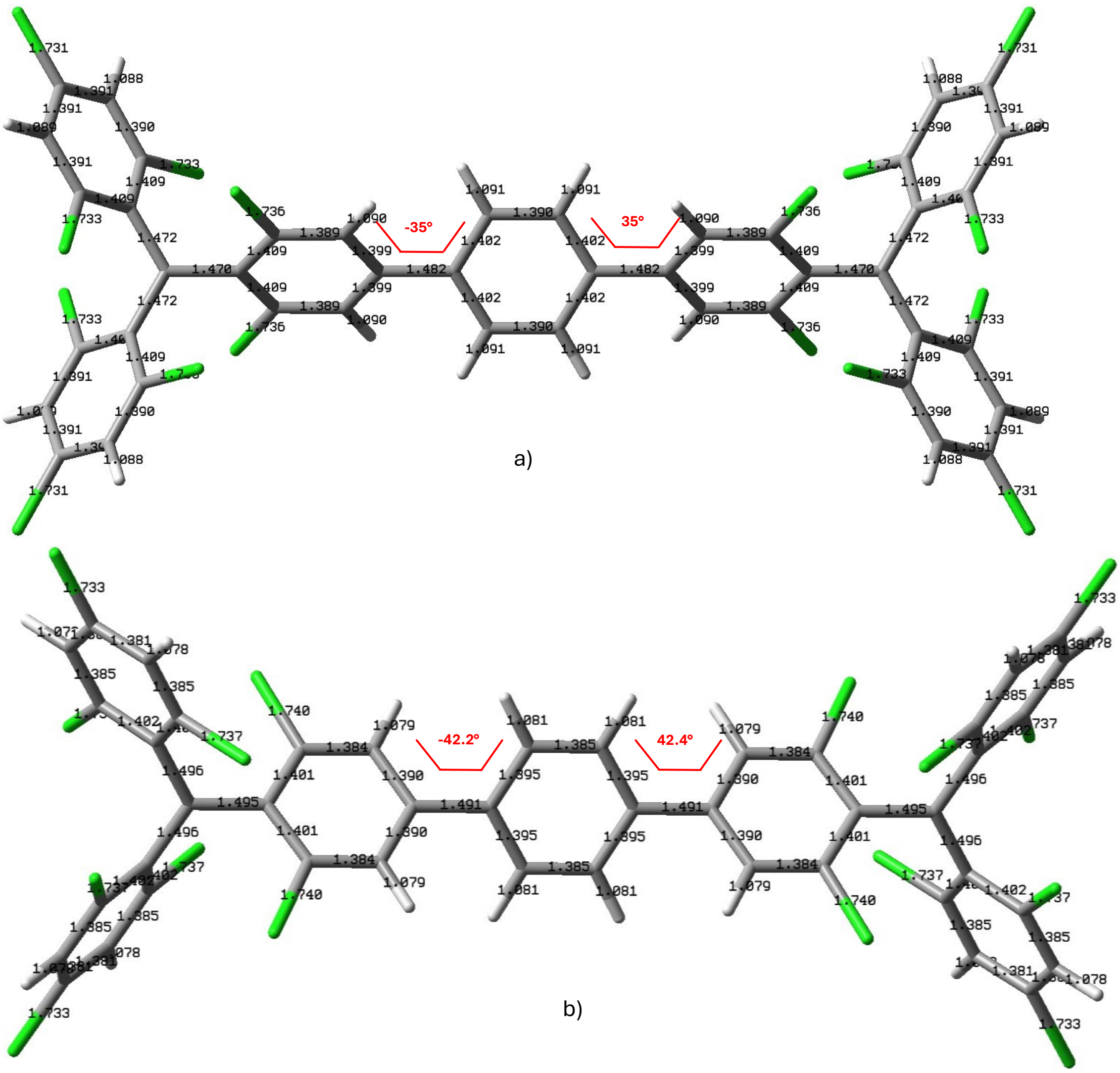

Figure S4. Computed bond lengths (Å) of the ground state of **TTM-ph-TTM** at (a) UM06-2X/def2SVP +D3 level and (b) CAS(4,3)/def2SVP level.

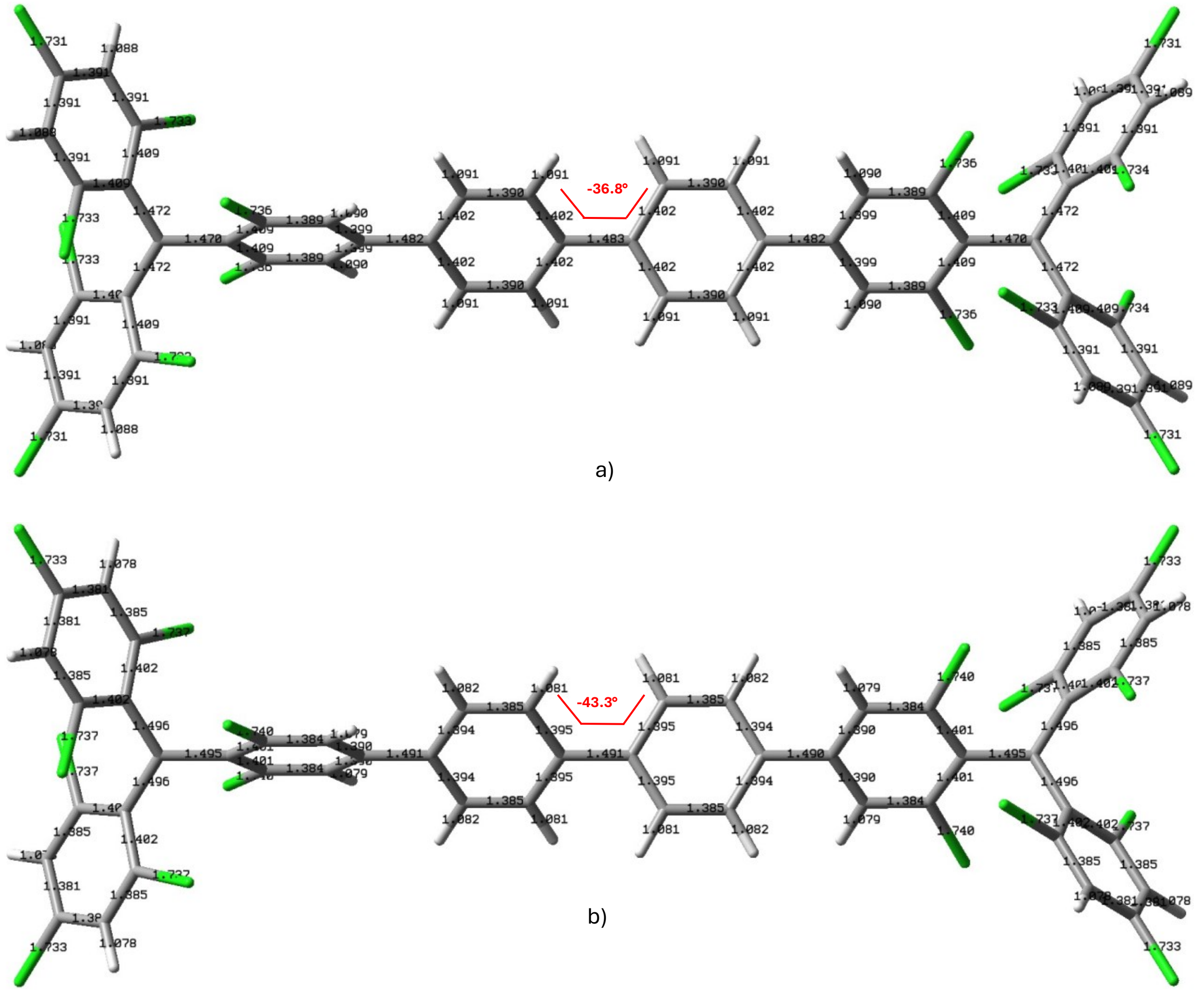

Figure S5. Computed bond lengths (Å) of the ground state of **TTM-ph-ph-TTM** at (a) UM06-2X/def2SVP +D3 level and (b) CAS(4,3)/def2SVP level.

2.1. Fragment orbital approach.

Compared to most conjugated diradicals in which the HOMO and LUMO delocalized orbitals (or the HOMO(α,β) and LUMO(α,β) localized set) play the most relevant role to identify lowest-lying excited states, the complete classification of low-lying excited state of **TTM**-derived diradicals must encompass a larger orbital space including the relevant fragment orbitals of each **TTM**-related unit (namely a set of almost degenerate occupied orbitals, the SOMO and a set of almost degenerate unoccupied orbitals) required to describe their essential photophysics. The fragment orbital analysis employed here expands each diradical MO as a linear combination of fragment orbitals, thereby allowing the identification of diradical excited states as linear combinations of local excitations of the fragments. The fragments have been chosen as half of the **TTM-TTM** and **TTM-ph-ph-TTM** diradicals. Thus, for **TTM-TTM** the reference radical fragment is **TTM-1H** namely **TTM** in which one Chlorine atom has been replaced by H. The **TTM fragment** corresponds to the **TTM-1H** radical without the H atom and was therefore described as a triplet state. For **TTM-ph-ph-TTM** the reference radical fragment is **TTM-ph-1H** namely **TTM** in which one Chlorine atom has been replaced by a phenyl group. The **TTM-ph fragment** corresponds to the **TTM-ph-1H** radical without the H atom and was therefore described as a triplet state. A comparison between the molecular orbitals of the reference radicals **TTM-1H**, **TTM-ph-1H** and the corresponding fragments is shown in Figures S6 and S7.

All the orbitals of each diradical were mapped into those of the corresponding fragments and expressed as linear combinations of fragment orbitals.

The molecular orbitals of the diradicaloid (DMOs) are expressed as linear combinations of the fragment molecular orbitals (FMOs) by the application of a projection operator, following the procedure outlined in previous work:[15–17]

$$|\psi_{Frag\_i}\rangle\langle\psi_{Frag\_i}|\psi_{Dirad\_j}\rangle = C_{i,j}^{Dirad\_FOB}|\psi_{Frag\_i}\rangle \quad (1)$$

Where $|\psi_{Frag\_i}\rangle$ are the molecular orbitals of the isolated fragments in the atomic orbital basis (AOB) and $|\psi_{Dirad\_j}\rangle$ are the molecular orbitals of the diradicaloid in the AOB. In matrix formulation $|\psi_{Frag\_i}\rangle$ form the column vectors of the $\boldsymbol{C}_{Frag\_AOB}$ matrix which is therefore a block diagonal matrix containing the MOs coefficients in the AOB from each fragment, with off block diagonals set to zero. Similarly, $|\psi_{Dirad\_j}\rangle$ form the column vectors of the $\boldsymbol{C}_{Dirad\_AOB}$ matrix.

The linear combinations describing DMOs in terms of FMOs are defined by the $C_{i,j}^{Dirad\_FOB}$ coefficients in the Fragment orbital basis (FOB) which, for a given aggregate orbital j, form the columns of the $\boldsymbol{C}_{Dirad\_FOB}$ matrix and are obtained as:

$$\boldsymbol{C}_{Dirad\_FOB} = \boldsymbol{C}_{Frag\_AOB}^{\boldsymbol{t}} \cdot \boldsymbol{S}_{Frag\_AOB} \cdot \boldsymbol{C}_{Dirad\_AOB} \quad (2)$$

where the $\boldsymbol{S}_{Frag\_AOB}$ is the overlap matrix of the fragments in the AOB and the superscript $\boldsymbol{t}$ indicates the transpose.

The overlap matrix $\boldsymbol{S}_{Dirad\_FOB}$ in the FOB is then required, to orthogonalize the fragment orbitals belonging to different fragments, using Löwdin's symmetric transformation[18]. The overlap matrix $\boldsymbol{S}_{Dirad\_FOB}$ between the FMOs is calculated from the MO coefficients of the fragment orbitals and the overlap of the atomic orbitals in the aggregate configuration $\boldsymbol{S}_{Dirad\_AOB}$.

$$\boldsymbol{S}_{Dirad\_FOB} = \boldsymbol{C}_{Frag\_AOB}^{\boldsymbol{t}} \cdot \boldsymbol{S}_{Dirad\_AOB} \cdot \boldsymbol{C}_{Frag\_AOB} \quad (3)$$

Finally, the aggregate's orbitals expressed in terms of orthogonalized monomer orbitals form the columns of the $\boldsymbol{C}^{L}_{Dirad\_FOB}$ matrix (superscript L indicates that Löwdin's orthogonalization has been applied) and are obtained as [15,16]

$$\boldsymbol{C}^{L}_{Dirad\_FOB} = \boldsymbol{S}^{-\frac{1}{2}}_{Dirad\_FOB} \cdot \boldsymbol{C}_{Dirad\_FOB} \tag{4}$$

The dimension of the $\boldsymbol{C}^{L}_{Dirad\_FOB}$ matrix corresponds to the full dimension of the FOB.

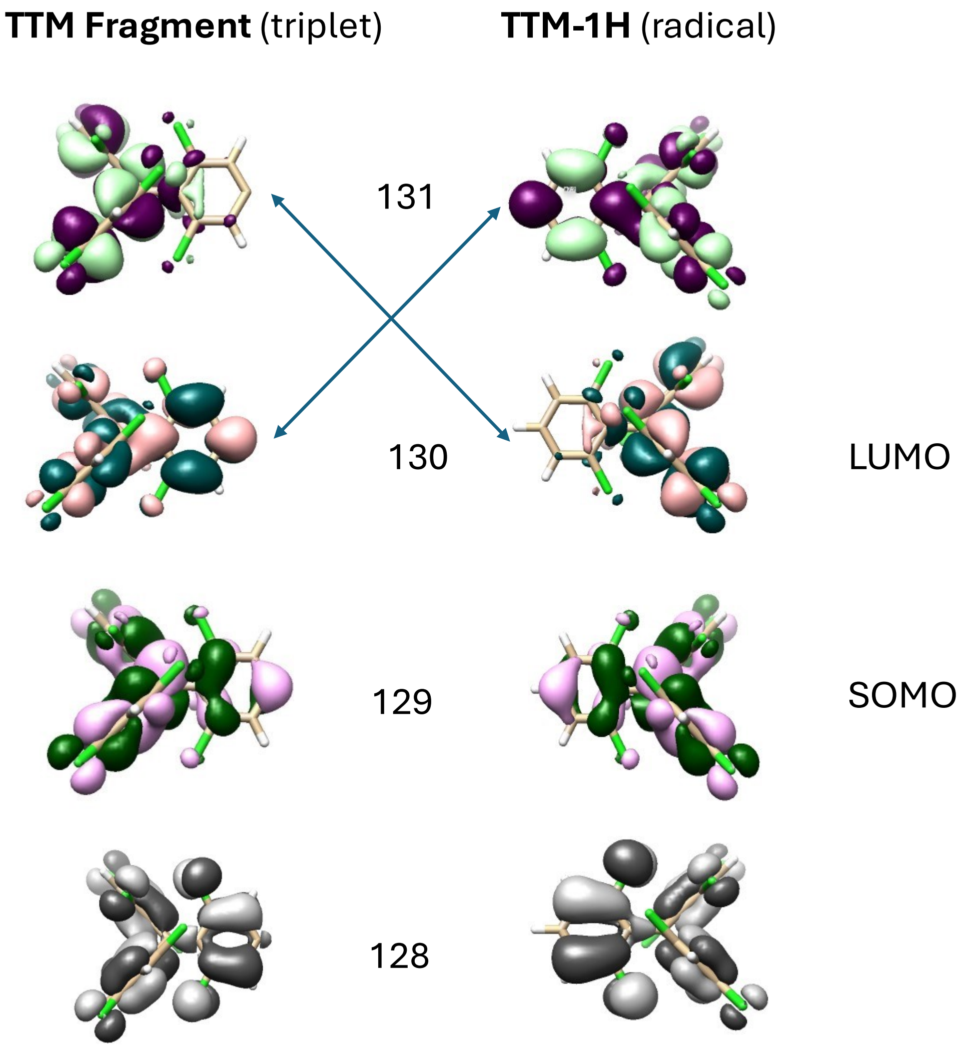

Figure S6. Comparison between orbitals (only α components are shown) of **TTM-1H** radical (right) and those of **TTM fragment** in the triplet state (left).

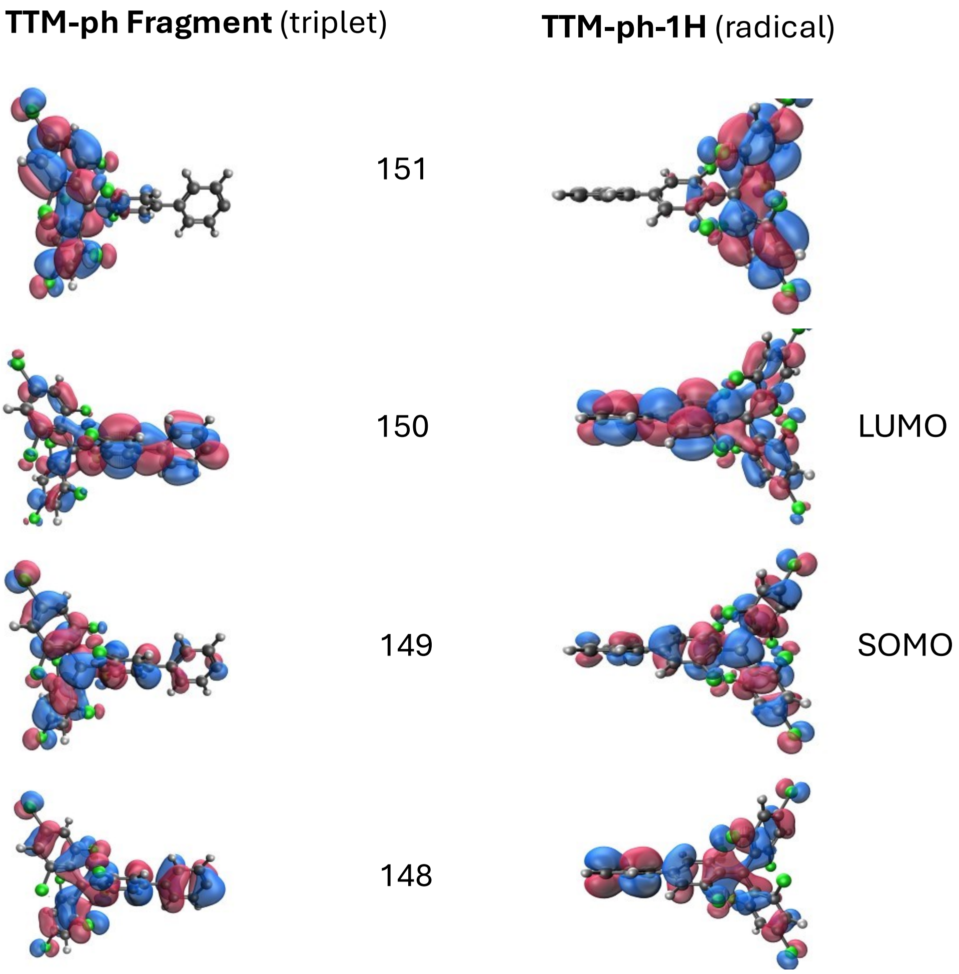

Figure S7. Comparison between orbitals (only α components are shown) of **TTM-ph-1H** radical (right) and those of **TTM-ph fragment** in the triplet state (left).

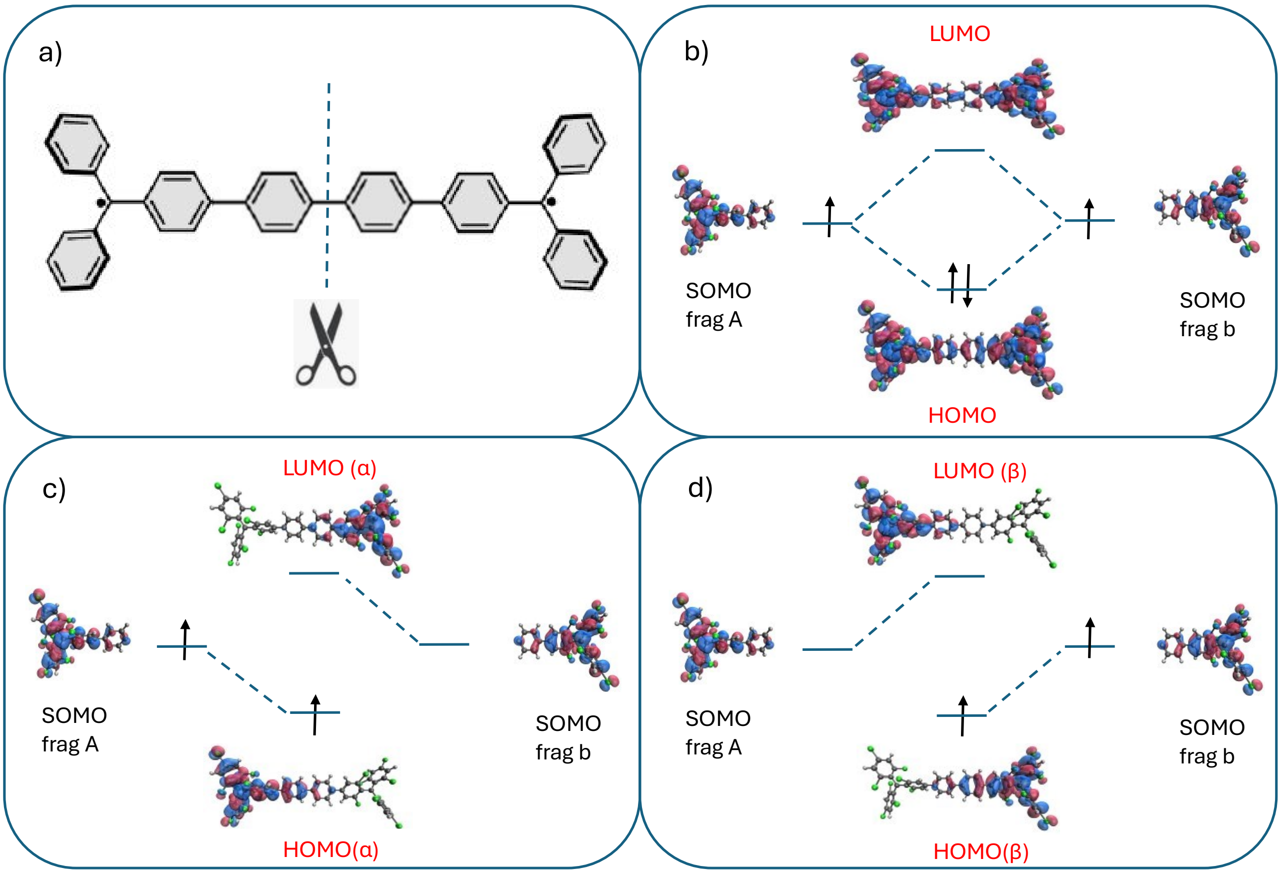

Figure S8. a) Schematic representation of the fragment orbital approach and interaction diagrams (in terms of **TTM-ph** SOMOs) for the formation of the MOs of **TTM-ph-ph-TTM**. b) Orbital interaction diagram for the HOMO and LUMO of **TTM-ph-ph-TTM** @ BS geometry (delocalized orbital basis). c) Orbital interaction diagram for the HOMO(α) and LUMO(α) of **TTM-ph-ph-TTM** @ BS geometry (localized orbital basis) and their relation with fragment orbitals. d) same as c) but for β electrons and orbitals

Table S4. Frontier orbitals of **TTM-TTM** and **TTM-ph-ph-TTM** expressed in terms of fragment orbitals ($SOMO_A$, $SOMO_B$). HOMO and LUMO are diradical orbitals computed at RB3LYP level at the ground state BS geometry (delocalized orbitals basis). HOMO(α) and LUMO(α) are diradical orbitals computed at UB3LYP level at the ground state BS geometry (localized orbitals basis). Only α components of the unrestricted orbitals are reported.

**TTM-TTM**				
Contribution from fragment orbital	HOMO	LUMO	HOMO(α)	LUMO(α)
$SOMO_A$	0.67	0.68	0.97	-0.06
$SOMO_B$	0.68	-0.68	0.04	0.91
**TTM-ph-ph-TTM**				
Contribution from fragment orbital	HOMO	LUMO	HOMO(α)	LUMO(α)
$SOMO_A$	0.68	0.69	-0.01	0.91
$SOMO_B$	0.68	-0.69	0.99	-0.01

2.2. Diradical character y_0.

The most common descriptor of diradical character is the y_0 index which assumes values between 0=no diradical character and 1=full diradical. y_0 can be determined at UHF level or using UDFT but the corresponding values can be rather different and functional dependent.[6] For this reason in this work the y_0 index was computed at PUHF/def2SVP level (at the computed equilibrium structures), following Yamaguchi's approach in the spin-projection scheme as[19]:

$$y_0^{PUHF} = 1 - \frac{2T_0}{1 + T_0^2} \quad (1)$$

with T_0 calculated as:

$$T_0 = \frac{n_{HONO} - n_{LUNO}}{2} \quad (2)$$

where n_{HONO} and n_{LUNO} are the occupation number of the highest occupied (HONO) and lowest unoccupied (LUNO) natural orbitals, respectively. All the above calculations were carried out with the Gaussian 16 package.[20] The computed y_0 values, for the investigated diradicaloids in their singlet ground electronic state are collected in Table S5. As previously reported, the y_0 values depend on the level of theory employed to compute them with y_0 (PUB3LYP) always smaller than y_0 (PUHF) values.

Table S5. Diradical character y_0 for the investigated diradicaloids computed at their BS optimized geometries.

Molecule[a]	$y_0(PUHF)$[b]	$y_0(PUB3LYP)$[c]
**TTH**[d]	0.32	
**TTM-TTM**	0.93	0.81
**TTM-ph-TTM**	0.98	0.94
**TTM-ph-ph-TTM**	0.99	0.98

[a]Optimized BS UM062X+D3 ground state geometry. [b]From UHF/def2SVP calculations. [c]From UB3LYP/def2SVP calculations. [d]Geometry optimized at RM062X/def2SVP calculations.

2.3. Identification of the SE and DE states from TDUDFT calculations.

When the localized orbital basis of UDFT calculations is employed for diradicals, we have previously shown that the lowest two excited states (DE and SE) are essentially dominated by the [HOMO(α) $\rightarrow$ LUMO(α)] $\pm$ [HOMO(β) $\rightarrow$ LUMO(β)] electronic excitations[5–7,21] where the +(–) signs refer to the DE dark (SE bright) states. These localized orbitals, given the large diradical character, essentially overlap with the SOMOs of one of the two radical building blocks. If we label A and B the two fragments as shown in Figure S9 for **TTM-TTM** and Figure S10 for **TTM-ph-ph-TTM**, then we have that HOMO(α) = LUMO(β) = $SOMO_A$ and HOMO(β) = LUMO(α) = $SOMO_B$. Thus, we can readily identify the lowest excited and emitting state of the investigated diradicals as the dipole forbidden DE state, a charge resonance state composed by the combination of the two charge transfer excitations: ($SOMO_A \rightarrow SOMO_B$) + ($SOMO_B \rightarrow SOMO_A$). The bright state responsible for the lowest strong absorption band is instead composed by the "minus" combination of the same two excitations.

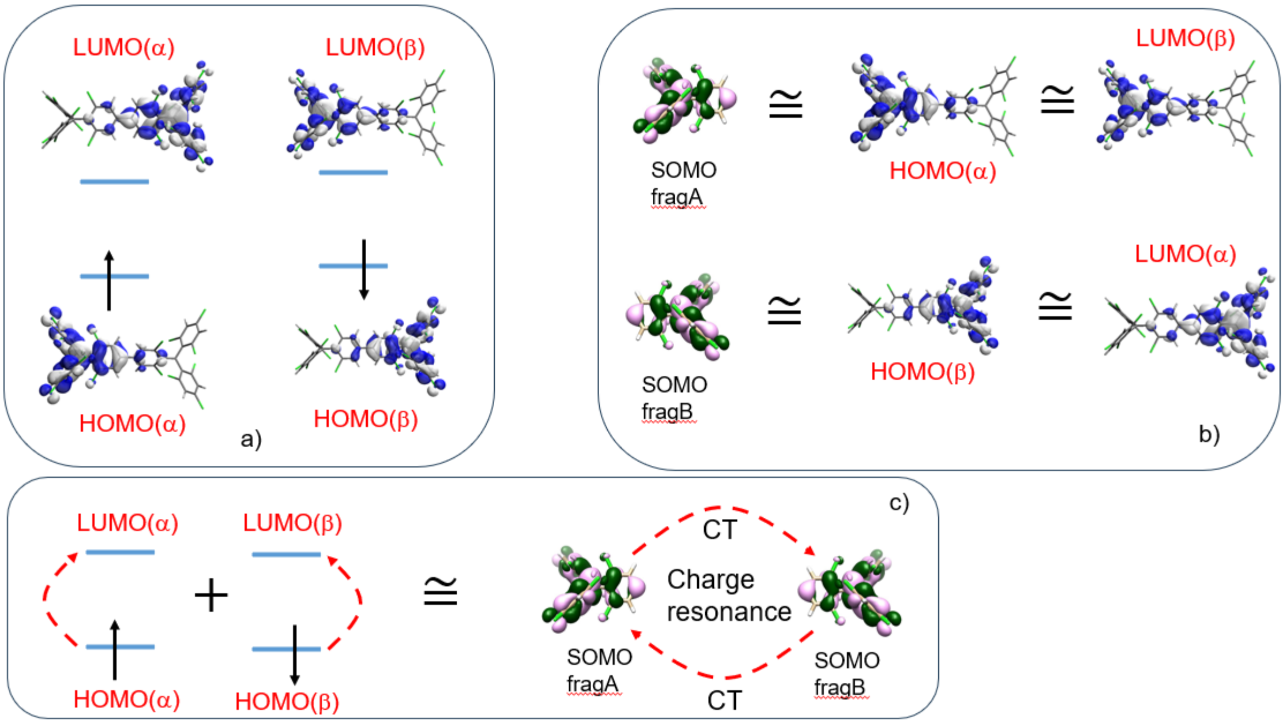

Figure S9. a) Localized frontier orbitals of **TTM-TTM** form UDFT calculations; b) similarity between localized diradical orbitals and **TTM** fragment SOMO orbitals; c) schematic representation of the charge resonance (i.e. combination of charge transfer excitations) nature of the DE emitting state in **TTM-TTM**.

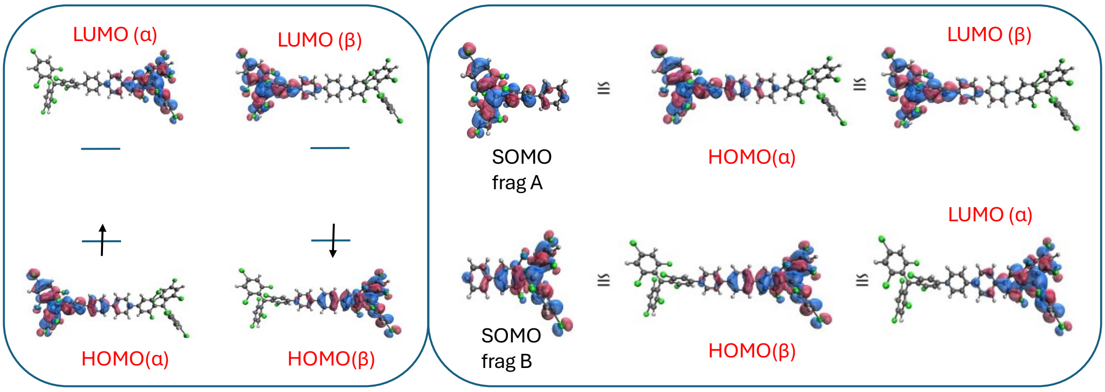

Figure S10. a) Localized frontier orbitals of **TTM-ph-ph-TTM** form UDFT calculations; b) similarity between localized diradical orbitals and **TTM-ph** fragment SOMO orbitals.

Excitation energies and oscillator strengths:

```
Excited State 1: 1.556  -A 2.0539 eV 603.66 nm f=0.0023 <S**2>=0.355
   255A ->257A      -0.14040
   256A ->257A 0.75106
   256A ->258A 0.10538
   255B ->257B 0.11913
   256B ->257B 0.60072
   256B ->258B      -0.10285
This state for optimization and/or second-order correction.
Total Energy, E(TD-HF/TD-DFT) =  -8815.27571278
Copying the excited state density for this state as the 1-particle RhoCI density.

Excited State 2: 1.394  -A 2.1611 eV 573.72 nm f=0.2024 <S**2>=0.236
   256A ->257A      -0.62404
   256B ->257B 0.77134

Excited State 3: 2.460  -A 2.7177 eV 456.22 nm f=0.0161 <S**2>=1.262
   250A ->257A      -0.18010
   251A ->257A      -0.23703
   253A ->257A 0.25630
   254A ->257A 0.82685
   256B ->259B 0.26901

Excited State 4: 2.450  -A 2.7297 eV 454.20 nm f=0.0064 <S**2>=1.250
   245A ->257A 0.14829
   246A ->257A 0.23916
   249A ->257A 0.10098
   252A ->257A 0.25335
   255A ->257A 0.69459
   255A ->258A 0.10240
   256A ->257A 0.18629
   256A ->258A      -0.15731
   255B ->257B      -0.23797
```

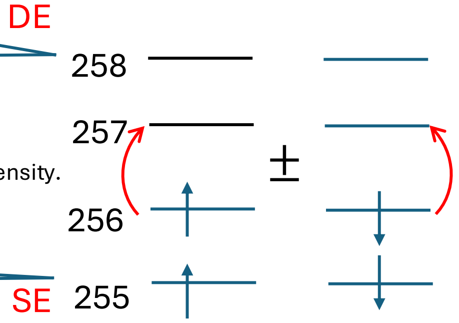

Figure S11. Lowest lying excited states of **TTM-TTM** from TDUB3LYP/def2SVP calculations @ optimized BS geometry (UM062X/def2SVP + D3). The ± combinations of the (256A→ 257A) and (256B→ 257B) excitations (enhanced in yellow) correspond to the DE and SE states, respectively.[5–7]

```
Excited State 1: 1.651  -A    2.1744 eV 570.20 nm f=0.0000 <S**2>=0.431
   275A ->277A 0.14982
   276A ->277A 0.67517
   276A ->278A 0.10316
   275B ->277B 0.14981
   276B ->277B 0.67431
   276B ->278B 0.10307
This state for optimization and/or second-order correction.
Total Energy, E(TD-HF/TD-DFT) =  -9046.17477777
Copying the excited state density for this state as the 1-particle RhoCI density.

Excited State 2: 1.452  -A 2.2182 eV 558.95 nm f=0.1138 <S**2>=0.277
   276A ->277A     -0.70042
   276B ->277B 0.70122

Excited State 3: 2.532  -A 2.6039 eV 476.15 nm f=0.0000 <S     **2>=1.353
   268A ->277A 0.13909
   272A ->277A 0.19457
   275A ->277A 0.49253
   275A ->278A 0.17690
   276A ->277A     -0.20671
   276A ->278A 0.26388
   268B ->277B 0.13963
   272B ->277B 0.19513
   275B ->277B 0.49420
   275B ->278B 0.17691
   276B ->277B     -0.20698
   276B ->278B 0.26314
```

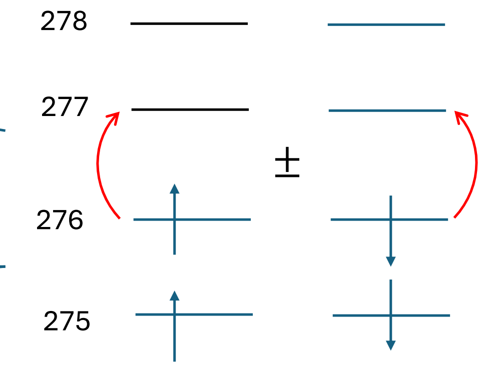

Figure S12. Lowest lying excited states of **TTM-ph-TTM** from TDUB3LYP/def2SVP calculations @ optimized BS geometry (UM062X/def2SVP + D3). The ± combinations of the (276A→ 277A) and (276B→ 277B) excitations (enhanced in yellow) correspond to the DE and SE states, respectively.[5–7]

```
Excited State 1: 1.661   -A 2.2386 eV 553.86 nm f=0.0002 <S**2>=0.439
   295A -> 297A 0.15366
   296A -> 297A 0.71270
   295B -> 297B     -0.14396
   296B -> 297B 0.63852
This state for optimization and/or second -order correction.
Total Energy, E(TD-HF/TD-DFT) =  -9277.07580000
Copying the excited state density for this state as the 1  -particle RhoCI density.

Excited State 2: 1.494   -A 2.2594 eV 548.75 nm f=0.0743 <S**2>=0.308
   296A -> 297A     -0.66148
   295B -> 297B     -0.10673
   296B -> 297B 0.73293

Excited State 3: 2.532   -A 2.5307 eV 489.93 nm f=0.0016 <S**2>=1.353
   292A -> 297A     -0.21691
   295A -> 297A     -0.43267
   295A -> 298A     -0.17800
   296A -> 297A 0.18997
   296A -> 298A     -0.24179
   296A -> 299A 0.12252
   283B -> 297B 0.10772
   292B -> 297B 0.27709
   295B -> 297B 0.55398
   295B -> 298B 0.18192
   296B -> 297B 0.21446
   296B -> 298B     -0.21092
```

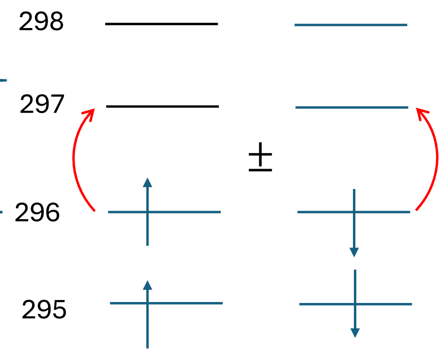

Figure S13. Lowest lying excited states of **TTM-ph-ph-TTM** from TDUB3LYP/def2SVP calculations @ optimized BS geometry (UM062X/def2SVP + D3). The ± combinations of the (296A→ 297A) and (296B→ 297B) excitations (enhanced in yellow) correspond to the DE and SE states, respectively.[5–7]

Table S6. Computed excitation energies E and oscillator strengths [f] for the **DE** and **SE** states of **TTM-TTM, TTM-ph-TTM, TTM-ph-ph-TTM** from TDUB3LYP/def2SVP and NEVPT2 calculations at the optimized BS geometry and comparison with observed absorption bands, when available.

Molecule @ geo BS	E(**DE** state) / eV [f]			E(**SE** state) / eV [f]		
	TDUB3LYP	NEVPT2	Exp Absorption	TDUB3LYP	NEVPT2	Exp Absorption
**TTM-TTM**	2.05 [0.002]	1.91 [0.000]	1.66	2.16 [0.202]	2.12 [0.309]	2.06
**TTM-ph-TTM**	2.17 [0.000]	2.14 [0.000]	Onset 2.07	2.22 [0.114]	2.27 [0.278]	2.21
**TTM-ph-ph-TTM**	2.24 [0.000]	2.33 [0.000]	-	2.26 [0.074]	2.38 [0.241]	-

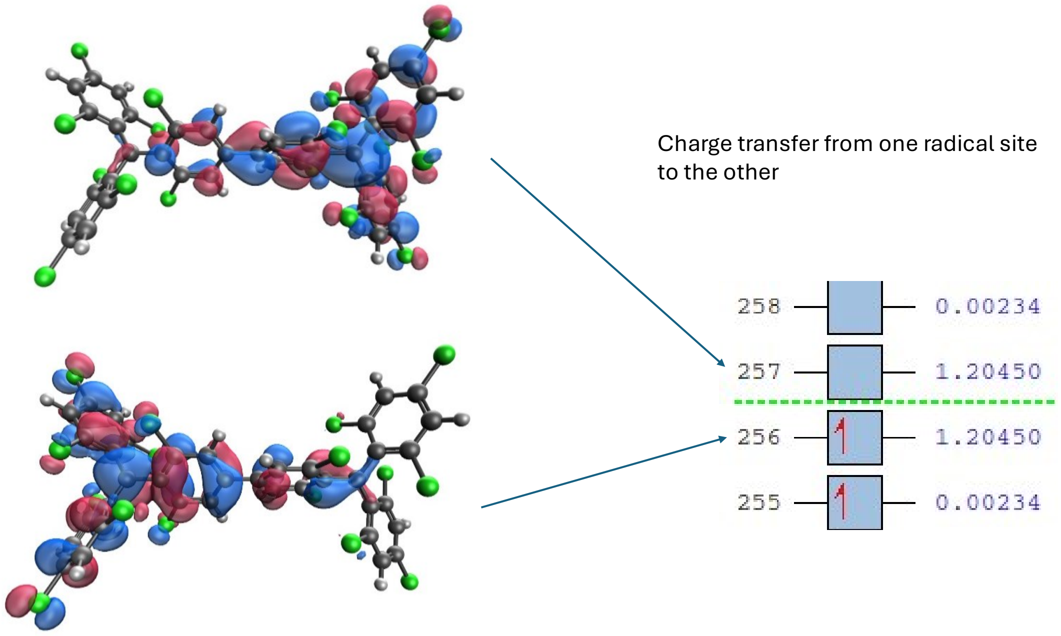

Figure S14. TTM-TTM: NTO analysis of the lowest excited state (the DE state) from TDUB3LYP/def2SVP calculations @ the BS optimized geometry, showing the charge transfer character. Plot of the highest occupied natural transition orbital (HONTO) and lowest unoccupied natural transition orbital (LUNTO). Only the α component is reported. An isodensity value of 0.05 has been used.

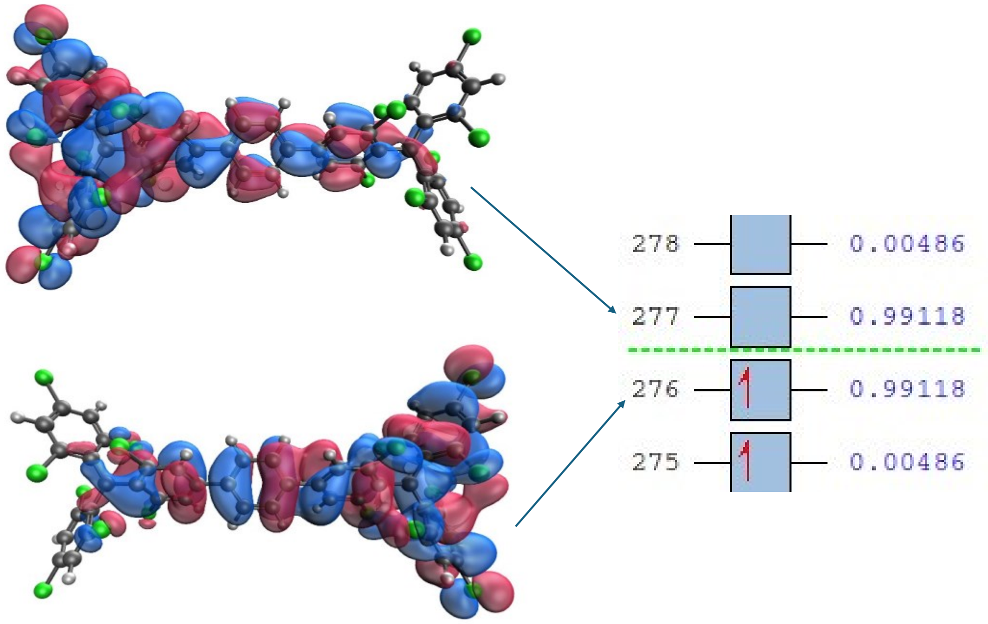

Figure S15. TTM-ph-TTM: NTO analysis of the lowest excited state (the DE state) from TDUB3LYP/def2SVP calculations @ the BS optimized geometry, showing the charge transfer character. Plot of the highest occupied natural transition orbital (HONTO) and lowest unoccupied natural transition orbital (LUNTO). Only the α component is reported. An isodensity value of 0.02 has been used.

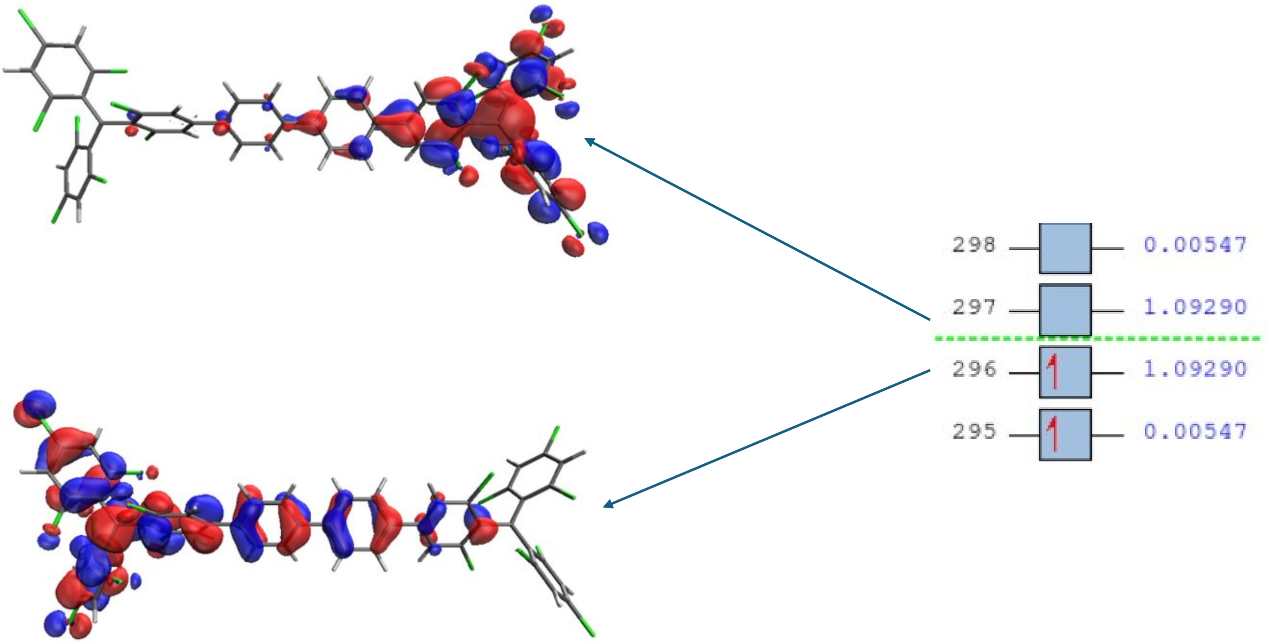

Figure S16. TTM-ph-ph-TTM: NTO analysis of the lowest excited state (the DE state) from TDUB3LYP/def2SVP calculations @ the BS optimized geometry, showing the charge transfer character. Plot of the highest occupied natural transition orbital (HONTO) and lowest unoccupied natural transition orbital (LUNTO). Only the α component is reported. An isodensity value of 0.05 has been used.

Table S7. TTM-TTM: TheoDORE[22] analysis of the TDUB3LYP/def2SVP excited states @ optimized BS geometry (UM062X/def2SVP + D3) with the identification of SE, DE and **TTM-**centred states.

state	dE(eV)	f	CT	
11.556-A	2.054	0.002	0.841	← CT state DE
21.394-A	2.161	0.203	0.929	← CT state SE
32.459-A	2.718	0.016	0.037	
42.450-A	2.730	0.006	0.359	
52.475-A	2.744	0.016	0.037	
62.455-A	2.761	0.030	0.193	← **TTM-**related state
72.393-A	2.950	0.004	0.042	
82.389-A	2.965	0.002	0.039	
92.505-A	2.982	0.000	0.148	
102.420-A	3.012	0.000	0.072	
112.496-A	3.031	0.000	0.088	
122.359-A	3.084	0.005	0.042	
132.481-A	3.092	0.002	0.174	
142.365-A	3.112	0.004	0.047	
152.436-A	3.204	0.519	0.411	← **TTM-**related state

Table S8. TTM-ph-TTM: TheoDORE[22] analysis of the TDUB3LYP/def2SVP excited states @ optimized BS geometry (UM062X/def2SVP + D3) with the identification of SE, DE and **TTM-**centred states.

state	dE(eV)	f	CT	
11.651-A	2.174	0.000	0.930	← CT state DE
21.452-A	2.218	0.114	0.983	← CT state SE
32.532-A	2.604	0.000	0.556	
42.422-A	2.640	0.070	0.256	
52.463-A	2.737	0.001	0.040	
62.464-A	2.737	0.028	0.040	← **TTM-**related state
72.402-A	2.933	0.000	0.043	
82.402-A	2.933	0.007	0.043	
92.617-A	2.945	0.000	0.164	
102.407-A	2.999	0.007	0.162	
112.492-A	3.031	0.076	0.182	← **TTM-**related state
122.468-A	3.031	0.015	0.106	
132.415-A	3.093	0.671	0.343	← **TTM-**related state
142.516-A	3.107	0.000	0.465	
152.362-A	3.109	0.000	0.037	
162.362-A	3.109	0.009	0.037	
172.641-A	3.361	0.000	0.055	
182.642-A	3.363	0.270	0.055	
192.716-A	3.411	0.001	0.452	
202.677-A	3.412	0.016	0.749	

Table S9. TTM-ph-ph-TTM: TheoDORE[22] analysis of the TDUB3LYP/def2SVP excited states @ optimized BS geometry (UM062X/def2SVP + D3) with the identification of SE, DE and **TTM-**centred states.

state	dE(eV)	f	CT	
11.661-A	2.239	0.000	0.913	← CT state DE
21.494-A	2.259	0.074	0.955	← CT state SE
32.532-A	2.531	0.002	0.373	
42.423-A	2.555	0.107	0.147	
52.463-A	2.737	0.013	0.008	
62.465-A	2.743	0.013	0.007	
72.780-A	2.910	0.000	0.165	
82.401-A	2.925	0.004	0.007	
92.403-A	2.927	0.004	0.007	
102.454-A	2.960	0.082	0.169	← **TTM-**related state
112.605-A	2.998	0.000	0.247	
122.504-A	3.000	0.421	0.123	← **TTM-**related state
132.481-A	3.047	0.002	0.050	
142.488-A	3.064	0.306	0.088	
152.362-A	3.112	0.004	0.007	
162.363-A	3.118	0.004	0.006	
172.713-A	3.220	0.000	0.336	
182.482-A	3.289	0.129	0.603	
192.666-A	3.354	0.000	0.454	
202.619-A	3.356	0.109	0.015	

Electron-hole correlation plots of the Omega matrices for the individual states.

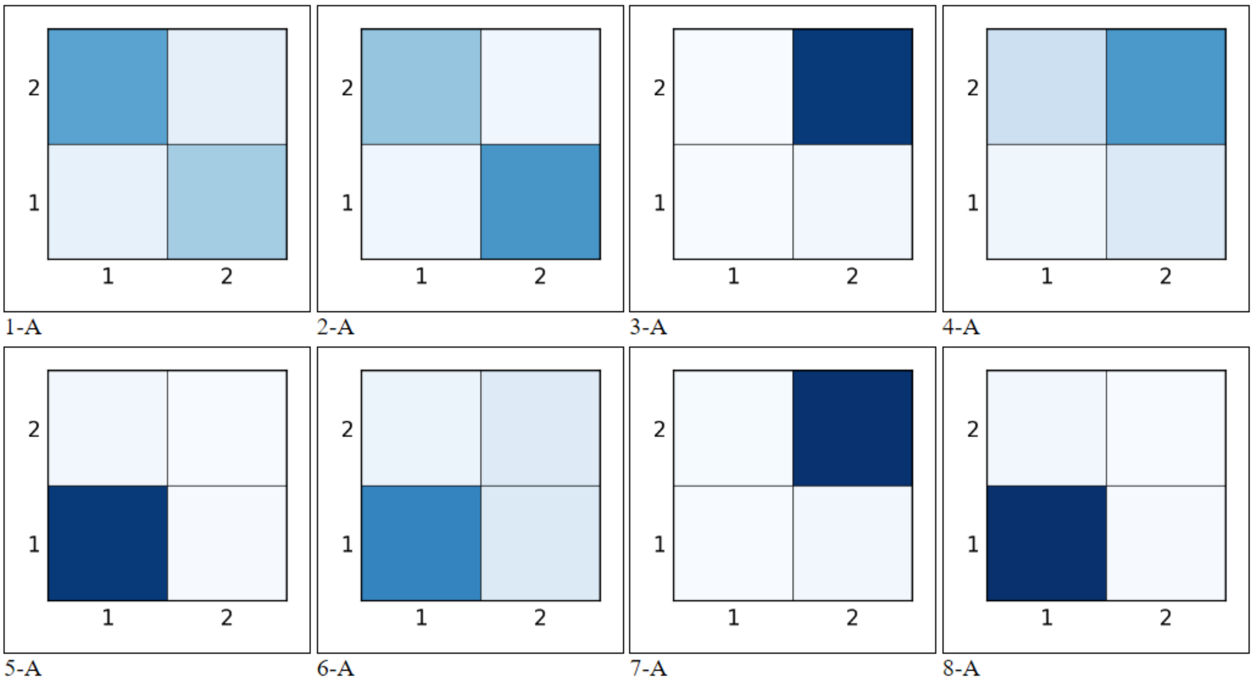

Figure S17. TTM-TTM: Electron-Hole plots from TheoDORE[22] analysis carried out on TDUB3LYP/def2SVP calculations. The local and charge transfer nature of the lowest 8 excited states is shown. Two fragments have been used: Fragment 1 and 2 correspond to terminal **TTM** radical moieties. Top left and bottom right medium or dark blue boxes denote CT states. Top right and bottom left medium or dark blue boxes identify local **TTM** radical centred-transitions.

Electron-hole correlation plots of the Omega matrices for the individual states.

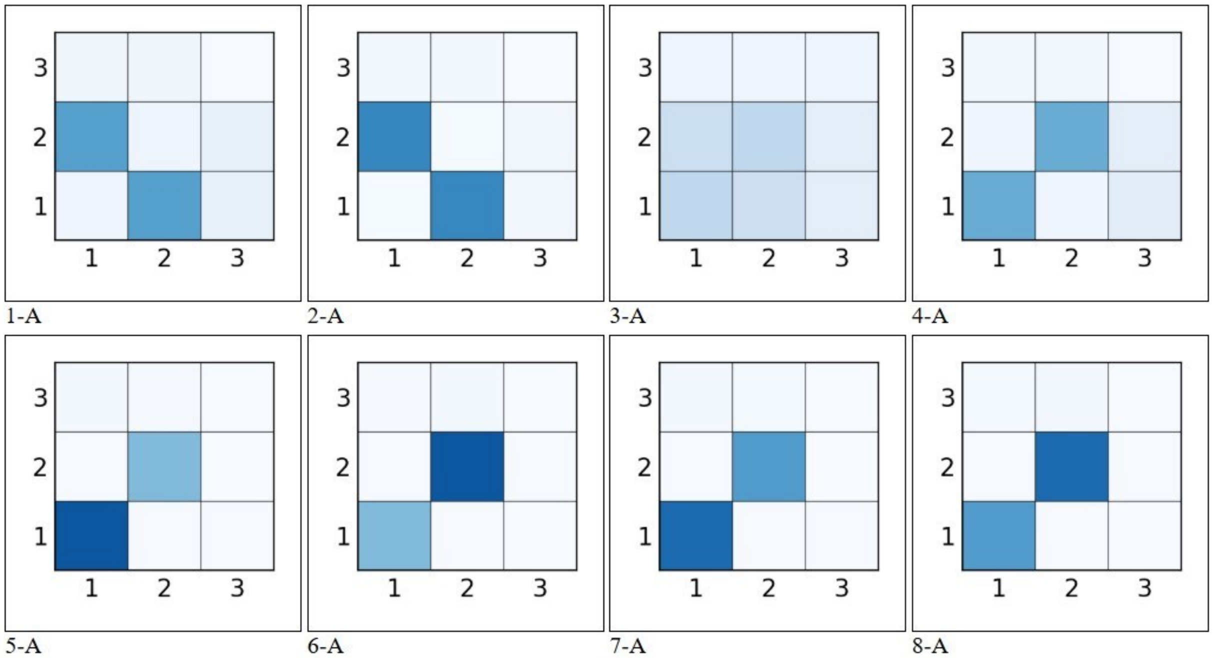

Figure S18. TTM-ph-TTM: Electron-Hole plots from TheoDORE[22] analysis carried out on TDUB3LYP/def2SVP calculations. The local and charge transfer nature of the lowest 8 excited states is shown. Three fragments have been used: Fragment 1 and 2 correspond to terminal **TTM** radical moieties, Fragment corresponds to the central phenyl ring. The lowest two states are of CT nature. States 4-8 are **TTM** local excitations.

Electron-hole correlation plots of the Omega matrices for the individual states.

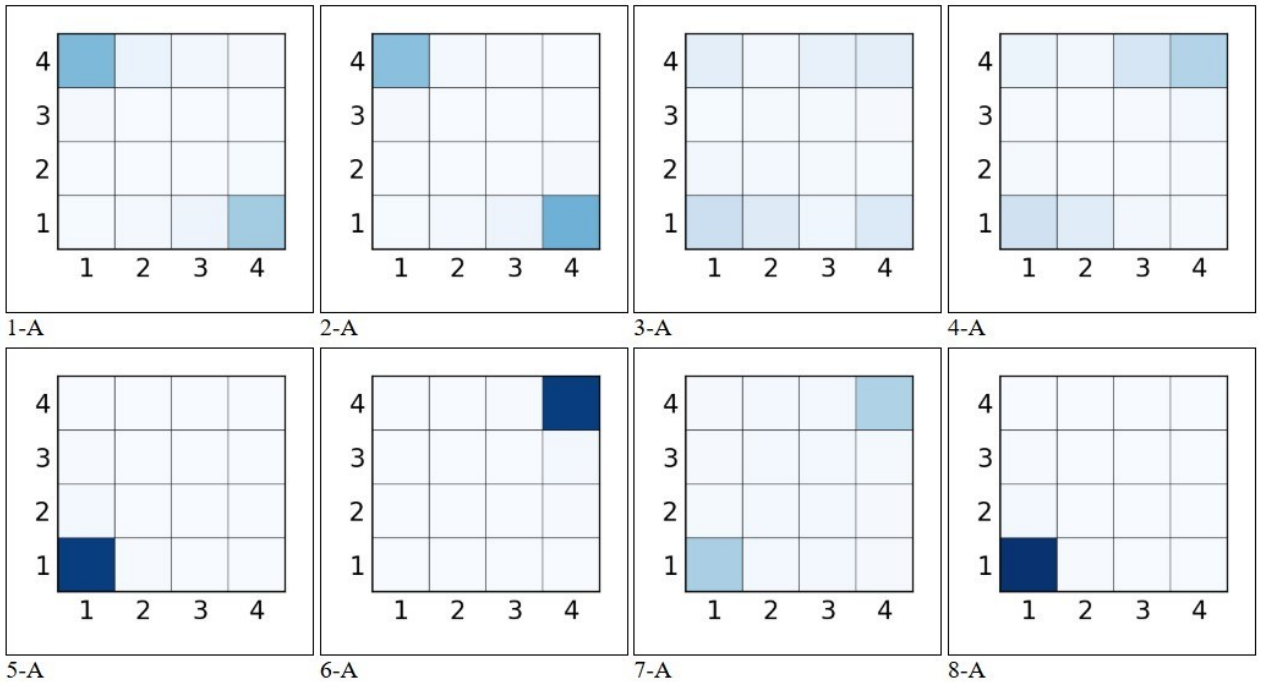

Figure S19. TTM-ph-ph-TTM: Electron-Hole plots from TheoDORE[22] analysis carried out on TDUB3LYP/def2SVP calculations. The local and charge transfer nature of the lowest 8 excited states is shown. Four fragments have been used in this case: Fragment 1 and 4 correspond to terminal **TTM** radical moieties, Fragment 2 and 3 correspond to the central phenyl rings. The lowest two states are of CT nature. States 5-8 are **TTM** local excitations.

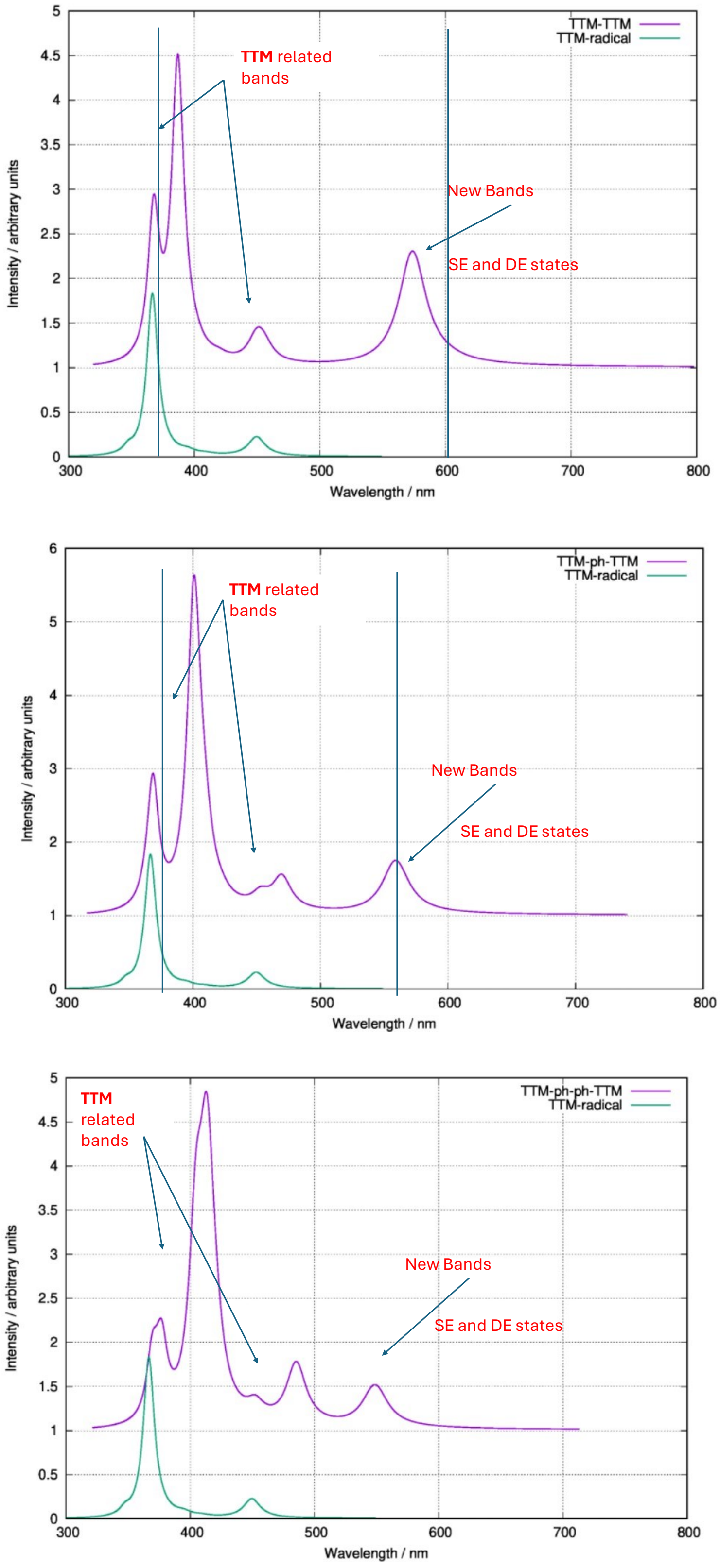

Figure S20. TDUB3LYP/def2SVP simulated absorption spectra of (top) **TTM-TTM**, (middle) **TTM-ph-TTM**, (bottom) **TTM-ph-ph-TTM** and comparison with the computed spectrum of **TTM** radical. The **TTM**-related bands and the new bands in the diradicals due to the SE and DE states are indicated. Blue vertical lines mark experimentally observed bands.

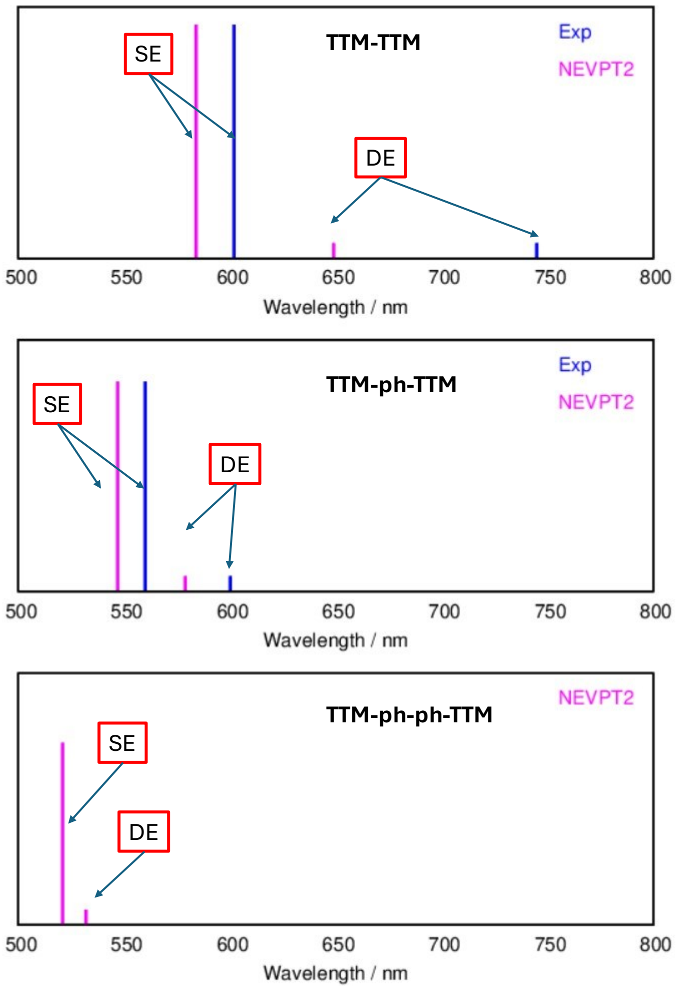

Figure S21. Proposed assignment of the transitions observed in absorption for **TTM-TTM** and **TTM-ph-TTM** (blue lines) based on the comparison with predicted transitions (from NEVPT2 calculations, magenta lines). Only computed results are reported for **TTM-ph-ph-TTM.** The intensities of the SE transitions are scaled according to the computed oscillator strengths. For the DE transition an arbitrary low intensity is attributed since the computed oscillator strength (and the observed absorption intensity) is negligible. The overestimation of the DE transition energy for **TTM-TTM** can be reconciled by considering that the computed value corresponds to the vertical excitation while the experimental band corresponds to the 0-0 adiabatic transition. Finally, the onset of the absorption spectrum of **TTM-ph-TTM** at ca. 600 nm[23] is assigned to the DE transition.

3. Experimental details

Solutions of **TTM-TTM** in 2-methyltetrahydrofuran (2-meTHF) were prepared in the dark at room temperature, also avoiding exposure to ambient light when handling the samples. The solvent, purchased from Sigma-Aldrich, was distilled, stored on molecular sieves and filtered before use. Low sample concentrations were used for emission measurements, corresponding to optical densities < 0.1. Higher concentrations (maintaining the optical density < 1) were used for absorption measurements. Stability of the solutions was checked by UV/vis spectroscopy and fluorescence spectroscopy; the samples, stored in the dark, were stable over a few days and did not decompose under the measurement conditions. Emission was measured with a FLS1000 fluorometer (Edinburgh

Instruments) equipped with a 450 W Xenon lamp, double grating excitation monochromator, automatic polarizers for anisotropy measurements, a PMT detector for the visible range and a nitrogen-cooled PMT detector for the near-IR range. Temperature was controlled by an OptistatDN (Oxford Instruments) cryostat operating with liquid nitrogen and fit into the sample chamber of the fluorometer, and an ITC601 (Oxford Instruments) temperature controller. The sample solution, contained in a 1 cm^2 quartz cuvette for cryogenics, was placed in the cryostat, cooled to the desired temperature and allowed to thermally equilibrate for a few minutes before spectral acquisition. Anisotropy was measured on a glassy 2-meTHF solution, obtained upon fast cooling of the sample down to 77 K. A long-pass optical filter was employed in emission to reject stray-light. Absorption spectra at room temperature were collected with a Perkin-Elmer Lambda 650 double beam UV/vis spectrophotometer. Absorption spectra at 77 K were collected in single-beam mode with the FLS1000 fluorometer on the sample vitrified into the OptistatDN cryostat, using a supplementary PMT detector collecting the transmitted light. The pure solvent, vitrified under the same experimental conditions, was used as reference.

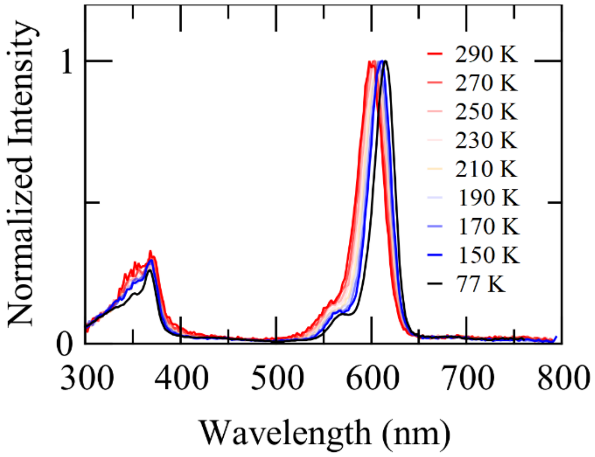

Figure S22. Excitation spectra of **TTM-TTM** in 2-meTHF at different temperatures (emission wavelength: 800 nm).

4. Cartesian coordinates of optimized geometries

TTM-TTM ground state BS geometry from UM062X/def2SVP + D3 calculations

Atom	X	Y	Z
Cl	-3.65666	-2.20572	-1.60145
C	-2.84409	-0.98744	-0.67108
C	-3.59223	0.00005	0.00006
C	-2.84405	0.98742	0.67134
Cl	-3.65653	2.20568	1.60183
C	-1.45529	0.99497	0.67608
H	-0.93770	1.79358	1.20689
C	-0.74135	-0.00005	0.00012
C	-1.45533	-0.99507	-0.67579
H	-0.93778	-1.79375	-1.20653
C	5.06162	0.00006	-0.00001
C	5.79846	-1.20927	-0.40083
C	5.54469	-2.46388	0.18772
Cl	4.40182	-2.59705	1.48441
C	6.23009	-3.61600	-0.18125
C	7.20893	-3.52825	-1.16610
Cl	8.07412	-4.95208	-1.63585
C	7.50300	-2.31455	-1.77944
C	6.79725	-1.18026	-1.39386
Cl	7.12263	0.29026	-2.25190
C	5.79859	1.20932	0.40078
C	6.79735	1.18017	1.39384
Cl	7.12242	-0.29038	2.25194
C	7.50331	2.31433	1.77942
C	7.20951	3.52807	1.16603
Cl	8.07496	4.95175	1.63577
C	6.23077	3.61597	0.18110
C	5.54517	2.46398	-0.18788
Cl	4.40259	2.59738	-1.48481
C	-5.06276	0.00009	-0.00001
C	-5.79872	1.20813	-0.40776
C	-5.54939	1.85226	-1.63560
Cl	-4.41569	1.17075	-2.75604
C	-6.23323	2.99556	-2.03382
C	-7.20718	3.52222	-1.19134
Cl	-8.07170	4.94246	-1.67304
C	-7.49719	2.92572	0.03176
C	-6.79196	1.78733	0.40614
Cl	-7.10958	1.13377	1.97996
C	-5.79868	-1.20802	0.40765

C	-6.79195	-1.78724	-0.40620
Cl	-7.10970	-1.13369	-1.98000
C	-7.49715	-2.92563	-0.03175
C	-7.20704	-3.52211	1.19134
Cl	-8.07153	-4.94235	1.67311
C	-6.23303	-2.99544	2.03374
C	-5.54923	-1.85213	1.63546
Cl	-4.41538	-1.17061	2.75574
H	6.01332	-4.56354	0.30842
H	8.25730	-2.25141	-2.56168
H	8.25755	2.25106	2.56170
H	6.01424	4.56352	-0.30866
H	-6.01941	3.45494	-2.99719
H	-8.24760	-3.34920	-0.69681
H	-6.01911	-3.45484	2.99708
H	-8.24759	3.34927	0.69688
C	1.45483	-0.42344	-1.12568
C	2.84341	-0.41601	-1.11915
C	3.59264	0.00002	0.00005
C	2.84343	0.41590	1.11932
C	1.45486	0.42329	1.12588
C	0.74032	-0.00006	0.00010
H	0.93765	-0.72004	-2.03786
Cl	3.65238	-0.84239	-2.59389
Cl	3.65239	0.84225	2.59408
H	0.93769	0.71985	2.03808

TTM-ph-TTM ground state BS geometry from UM062X/def2SVP + D3 calculations

Nato	X	Y	Z
Cl	-5.81388	1.59847	2.20776
C	-5.00201	0.73840	0.93723
C	-5.75109	-0.00009	-0.00007
C	-5.00201	-0.73859	-0.93736
Cl	-5.81386	-1.59861	-2.20794
C	-3.61329	-0.74603	-0.94263
H	-3.09604	-1.30663	-1.72082
C	-2.89695	-0.00011	-0.00005
C	-3.61329	0.74582	0.94252
H	-3.09604	1.30642	1.72071
C	7.22149	-0.00001	0.00002
C	7.95671	1.27504	-0.01227
C	7.70340	2.27020	-0.97682
Cl	6.57038	1.96998	-2.25380
C	8.38250	3.48330	-0.99699
C	9.35526	3.72399	-0.03198
Cl	10.21123	5.22863	-0.04401
C	9.65024	2.77522	0.94200
C	8.95036	1.57362	0.94068
Cl	9.27518	0.46015	2.22876
C	7.95679	-1.27502	0.01229
C	8.95043	-1.57354	-0.94068
Cl	9.27515	-0.46008	-2.22879
C	9.65039	-2.77510	-0.94201
C	9.35549	-3.72388	0.03198
Cl	10.21157	-5.22846	0.04401
C	8.38275	-3.48325	0.99702
C	7.70357	-2.27019	0.97686
Cl	6.57057	-1.97003	2.25388
C	-7.22144	-0.00003	-0.00007
C	-7.95685	-1.27500	0.01018
C	-7.70368	-2.27175	0.97312
Cl	-6.57051	-1.97373	2.25048
C	-8.38294	-3.48479	0.99131
C	-9.35570	-3.72379	0.02588
Cl	-10.21183	-5.22836	0.03537
C	-9.65054	-2.77340	-0.94655
C	-8.95050	-1.57190	-0.94327
Cl	-9.27508	-0.45639	-2.22964
C	-7.95672	1.27501	-0.01022
C	-8.95020	1.57203	0.94338
Cl	-9.27472	0.45657	2.22981
C	-9.65011	2.77361	0.94675
C	-9.35530	3.72396	-0.02573
Cl	-10.21124	5.22863	-0.03510
C	-8.38270	3.48485	-0.99130
C	-7.70357	2.27173	-0.97320
Cl	-6.57064	1.97358	-2.25074

H	8.16571	4.22017	-1.76817
H	10.40014	2.97221	1.70613
H	10.40028	-2.97205	-1.70616
H	8.16603	-4.22012	1.76821
H	-8.16622	-4.22296	1.76127
H	-10.39985	2.96937	1.71134
H	-8.16601	4.22299	-1.76129
H	-10.40042	-2.96907	-1.71104
C	3.61331	0.74697	0.94167
C	5.00203	0.73961	0.93636
C	5.75112	-0.00004	0.00001
C	5.00206	-0.73971	-0.93636
C	3.61334	-0.74711	-0.94169
C	2.89695	-0.00008	-0.00002
H	3.09611	1.30846	1.71924
Cl	5.81394	1.60126	2.20580
Cl	5.81402	-1.60136	-2.20578
H	3.09617	-1.30863	-1.71926
C	0.69498	-1.15340	-0.34226
C	-0.69499	-1.15340	-0.34230
C	-1.41453	-0.00011	-0.00005
C	-0.69500	1.15319	0.34222
C	0.69496	1.15321	0.34219
C	1.41453	-0.00010	-0.00003
H	1.23107	-2.07398	-0.57906
H	-1.23111	-2.07395	-0.57919
H	-1.23114	2.07372	0.57910
H	1.23104	2.07379	0.57901

TTM-ph-ph-TTM ground state BS geometry from UM062X/def2SVP + D3 calculations

Nato	X	Y	Z
Cl	-7.97238	0.81456	2.60175
C	-7.16083	0.40230	1.12321
C	-7.91023	0.00001	0.00001
C	-7.16084	-0.40227	-1.12320
Cl	-7.97241	-0.81455	-2.60173
C	-5.77214	-0.40773	-1.13061
H	-5.25472	-0.69239	-2.04641
C	-5.05538	0.00003	-0.00001
C	-5.77213	0.40778	1.13060
H	-5.25471	0.69244	2.04640
C	9.38090	-0.00001	0.00002
C	10.11758	-1.19869	0.43306
C	9.86806	-1.81805	1.67359
Cl	8.73302	-1.11465	2.77889
C	10.55255	-2.95262	2.09528
C	11.52727	-3.49557	1.26417
Cl	12.39251	-4.90565	1.77459
C	11.81759	-2.92380	0.02946
C	11.11164	-1.79377	-0.36826
Cl	11.42999	-1.17244	-1.95505
C	10.11760	1.19866	-0.43302
C	11.11157	1.79383	0.36836
Cl	11.42976	1.17267	1.95525
C	11.81754	2.92384	-0.02937
C	11.52734	3.49552	-1.26415
Cl	12.39261	4.90558	-1.77458
C	10.55273	2.95248	-2.09532
C	9.86821	1.81793	-1.67362
Cl	8.73333	1.11442	-2.77902
C	-9.38028	-0.00000	0.00002
C	-10.11720	-1.20976	-0.39979
C	-9.86618	-2.46372	0.19143
Cl	-8.72779	-2.59555	1.49196
C	-10.55150	-3.61600	-0.17749
C	-11.52766	-3.52923	-1.16494
Cl	-12.39288	-4.95356	-1.63459
C	-11.81936	-2.31638	-1.78088
C	-11.11359	-1.18202	-1.39528
Cl	-11.43695	0.28773	-2.25560
C	-10.11722	1.20976	0.39980
C	-11.11364	1.18201	1.39526
Cl	-11.43703	-0.28775	2.25555
C	-11.81943	2.31637	1.78084
C	-11.52771	3.52922	1.16491
Cl	-12.39294	4.95355	1.63454
C	-10.55151	3.61599	0.17750

C	-9.86618	2.46371	-0.19140
Cl	-8.72773	2.59554	-1.49188
H	10.33856	-3.39237	3.06777
H	12.56869	-3.36021	-0.62652
H	12.56855	3.36032	0.62667
H	10.33883	3.39216	-3.06788
H	-10.33681	-4.56273	0.31462
H	-12.57189	2.25379	2.56486
H	-10.33680	4.56273	-0.31460
H	-12.57180	-2.25381	-2.56493
C	5.77269	-1.01507	-0.64343
C	7.16135	-1.00673	-0.64024
C	7.91055	0.00000	-0.00000
C	7.16136	1.00674	0.64022
C	5.77269	1.01510	0.64340
C	5.05577	0.00002	-0.00002
H	5.25560	-1.80112	-1.19298
Cl	7.97398	-2.23897	-1.55433
Cl	7.97399	2.23898	1.55430
H	5.25560	1.80116	1.19294
C	2.85327	1.20157	-0.05076
C	1.46313	1.20116	-0.05075
C	0.74164	0.00003	-0.00003
C	1.46312	-1.20110	0.05070
C	2.85326	-1.20153	0.05072
C	3.57333	0.00002	-0.00002
H	3.38913	2.14937	-0.12619
H	0.92666	2.14861	-0.12556
H	0.92664	-2.14855	0.12551
H	3.38912	-2.14933	0.12615
C	-1.46301	-0.99190	-0.67928
C	-2.85318	-0.99128	-0.68107
C	-3.57291	0.00003	-0.00001
C	-2.85317	0.99135	0.68104
C	-1.46301	0.99196	0.67923
C	-0.74167	0.00003	-0.00002
H	-0.92612	-1.76646	-1.22963
H	-3.38970	-1.78388	-1.20561
H	-3.38970	1.78394	1.20558
H	-0.92611	1.76653	1.22959

References

[1] M. Ballester, J. Riera, J. Castañer, C. Rovira, O. Armet, *Synth.* **1986**, *1986*, 64–66.

[2] Q. Peng, A. Obolda, M. Zhang, F. Li, *Angew. Chemie Int. Ed.* **2015**, *54*, 7091–7095.

[3] S. Grimme, J. Antony, S. Ehrlich, H. Krieg, *J. Chem. Phys.* **2010**, *132*, 154104.

[4] S. Grimme, S. Ehrlich, L. Goerigk, *J. Comput. Chem.* **2011**, *32*, 1456–1465.

[5] S. Canola, Y. Dai, F. Negri, *Computation* **2019**, *7*, 68.

[6] S. Canola, J. Casado, F. Negri, *Phys. Chem. Chem. Phys.* **2018**, *20*, 24227–24238.

[7] F. Negri, S. Canola, Y. Dai, in *Diradicaloids* (Ed.: J. Wu), Jenny Stanford Publishing, New York, **2022**, pp. 145–179.

[8] B. 0 Roos, P. R. Taylor, P. E. M. Siegbahn, *Chem. Phys.* **1980**, *48*.

[9] C. Angeli, R. Cimiraglia, S. Evangelisti, T. Leininger, J. P. Malrieu, *J. Chem. Phys.* **2001**, *114*, DOI 10.1063/1.1361246.

[10] C. Angeli, R. Cimiraglia, J. P. Malrieu, *J. Chem. Phys.* **2002**, *117*, DOI 10.1063/1.1515317.

[11] F. Neese, *Wiley Interdiscip. Rev. Comput. Mol. Sci.* **2012**, *2*, 73–78.

[12] F. Neese, F. Wennmohs, U. Becker, C. Riplinger, *J. Chem. Phys.* **2020**, *152*, 224108.

[13] A. Schäfer, H. Horn, R. Ahlrichs, *J. Chem. Phys.* **1998**, *97*, 2571.

[14] F. Weigend, *J. Comput. Chem.* **2008**, *29*, 167–175.

[15] J. E. Norton, J.-L. Brédas, *J. Chem. Phys.* **2008**, *128*, 034701.

[16] D. Kim, *Bull. Korean Chem. Soc.* **2015**, *36*, 2284–2289.

[17] S. Canola, G. Bagnara, Y. Dai, G. Ricci, A. Calzolari, F. Negri, *J. Chem. Phys.* **2021**, *154*, 124101.

[18] P. Löwdin, *J. Chem. Phys.* **1950**, *18*, 365–375.

[19] K. Yamaguchi, *Chem. Phys. Lett.* **1975**, *33*, 330–335.

[20] M. J. Frisch, G. W. Trucks, H. B. Schlegel, G. E. Scuseria, M. A. Robb, J. R. Cheeseman, G. Scalmani, V. Barone, G. A. Petersson, H. Nakatsuji, X. Li, M. Caricato, A. V. Marenich, J. Bloino, B. G. Janesko, R. Gomperts, B. Mennucci, H. P. Hratchian, J. V. Ortiz, A. F. Izmaylov, J. L. Sonnenberg, D. Williams-Young, F. Ding, F. Lipparini, F. Egidi, J. Goings, B. Peng, A. Petrone, T. Henderson, D. Ranasinghe, J. Zakrzewski, V. G.; Gao, N. Rega, G. Zheng, W. Liang, M. Hada, M. Ehara, K. Toyota, R. Fukuda, J. Hasegawa, M. Ishida, T. Nakajima, Y. Honda, O. Kitao, H. Nakai, T. Vreven, K. Throssell, J. Montgomery, J. A., J. E. Peralta, F. Ogliaro, M. J. Bearpark, J. J. Heyd, E. N. Brothers, K. N. Kudin, V. N. Staroverov, T. A. Keith, R. Kobayashi, J. Normand, K. Raghavachari, A. P. Rendell, J. C. Burant, S. S. Iyengar, J. Tomasi, M. Cossi, J. M. Millam, M. Klene, C. Adamo, R. Cammi, J. W. Ochterski, R. L. Martin, K. Morokuma, O. Farkas, J. B. Foresman, D. J. Fox, *Revis. A.03, Gaussian, Inc., Wallingford CT,* **2016**.

[21] V. Bonačić-Koutecký, J. Koutecký, J. Michl, *Angew. Chemie Int. Ed. English* **1987**, *26*, 170–189.

[22] F. Plasser, *J. Chem. Phys.* **2020**, *152*, DOI 10.1063/1.5143076.

[23] A. Abdurahman, J. Wang, Y. Zhao, P. Li, L. Shen, Q. Peng, *Angew. Chem. Int. Ed. Engl.* **2023**, *62*, e202300772.
